# Suppressing non-radiative recombination in metal halide perovskite solar cells by synergistic effect of ferroelasticity

**DOI:** 10.1038/s41467-023-35837-1

**Published:** 2023-01-17

**Authors:** Wei Qin, Wajid Ali, Jianfeng Wang, Yong Liu, Xiaolan Yan, Pengfei Zhang, Zhaochi Feng, Hao Tian, Yanfeng Yin, Wenming Tian, Can Li

**Affiliations:** 1 State Key Laboratory of Catalysis, Dalian National Laboratory for Clean Energy, Dalian Institute of Chemical Physics, Chinese Academy of Sciences, Dalian, 116023 China; 2 University of Chinese Academy of Sciences, Beijing, 100049 China; 3 School of Physics, Beihang University, Beijing, 100191 China; 4 Beijing Computational Science Research Center, Beijing, 100193 China; 5 State key laboratory of molecular reaction dynamics and the dynamic research center for energy and environmental materials, Dalian Institute of Chemical Physics, Chinese Academy of Sciences, Dalian, 116023 China

**Keywords:** Solar cells, Solar cells, Ferroelectrics and multiferroics

## Abstract

The low fraction of non-radiative recombination established the foundation of metal halide perovskite solar cells. However, the origin of low non-radiative recombination in metal halide perovskite materials is still not well-understood. Herein, we find that the non-radiative recombination in twinning-tetragonal phase methylammonium lead halide (MAPbI_x_Cl_3-x_) is apparently suppressed by applying an electric field, which leads to a remarkable increase of the open-circuit voltage from 1.12 V to 1.26 V. Possible effects of ionic migration and light soaking on the open-circuit voltage enhancement are excluded experimentally by control experiments. Microscopic and macroscopic characterizations reveal an excellent correlation between the ferroelastic lattice deformation and the suppression of non-radiative recombination. The calculation result suggests the existence of lattice polarization in self-stabilizable deformed domain walls, indicating the charge separation that facilitated by lattice polarization is accountable for the suppressed non-radiative recombination. This work provides an understanding of the excellent performance of metal halide perovskite solar cells.

## Abstract (web summary)

The origin of low non-radiative recombination in perovskites was not well-understood. Here, authors apply electric field to manipulate ferroelastic lattice deformation to study non-radiative recombination and strain in twinning tetragonal phase, achieving an increased open-circuit voltage to 1.26 V.

## Introduction

Metal halide perovskite (MHP) analogous solar cells have opened up a new avenue for the next generation photovoltaic technologies^1–3^, as these materials show high light absorption coefficient, band-like transport of charge carriers, long lifetime of charge carrier, and especially the low fraction of non-radiative recombination^4^. The latter established the foundation of photovoltaic applications of MHP materials since the ideal solar cell operates at its radiative limits. Nowadays, theories and approaches for suppression of non-radiative recombination are mainly concentrated on eliminating recombination pathways (i.e., defect passivation^5,6^ and interfacial engineering^7,8^) and constructing graded junction^9^ (similar to the functionality of that p-n junctions in cascaded device structure enhances carrier injection^10,11^). Those strategies are aiming at understanding and solving issues at the device scale, hence the origin of the low non-radiative recombination in MHP materials is still under debate.

One alternative explanation that has been proposed is the ferroic property-induced lattice polarization that may facilitate spatial charge separation and the non-radiative recombination is thereafter suppressed^12^. As an analogy, it was reported that the lattice polarization in inorganic perovskite materials facilitates charge separation^12,13^, and even enables an open-circuit voltage (*V*_oc_) higher than the energy bandgap^14,15^. It seems that lattice polarization plays a vital role in reducing non-radiative recombination. And two factors are critical for the study of lattice polarization in MHP material: the non-centrosymmetric unit cells and the ferroelasticity of the crystallographic structures.

It is well known that 20 out of 21 non-centrosymmetric crystal classes can form deformative lattice structures with charge displacement resulting in piezoelectric lattice polarization^16^. Methylammonium lead halide (MAPbI_x_Cl_3-x_) exhibits a centrosymmetric lattice structure (cubic, ${Pm}\bar{3}m$, C-phase) at a temperature above ~330 K^17^ which is lower than the annealing temperature for crystallization. In this regard, it is anticipated that the spontaneous phase transition is associated with the annealing-cooling post-treatment. During the post-annealing cooling, PbI_3_^−^ octahedral rotates around the c-axial and triggers the expansion of the I-Pb-I bond along the c-axis. The crystallographic structure of MAPbI_x_Cl_3-x_ is subsequently transformed to a lower symmetry phase (tetragonal, T-phase)^18^. But here, the space group of T-phase is still under debate for the assignment to either $I4/{mcm}$ (centrosymmetry) or $I4{cm}$ (non-centrosymmetry). The contradiction is originated from the structural flexibility of MHP materials showing remarkable positional freedom of organic cation and anionic movement^18^. Therefore, there is no clear consensus on the existence of lattice polarization in MHP materials.

Domain structure is commonly formed as the responsivity of the lattice to minimize the elastic strain energy^19^. For MAPbI_x_Cl_3-x_, the existence of ferroelastic twin-domains has been confirmed by both local^20–23^ and non-local probes^2,24,25^. Liu et al. revealed the discrepancy between elastic variation and segregation of chemical composition within twin-domains^20^. Kennard et al. further confirmed the existence of strain-stress retention in MAPbI_x_Cl_3-x_ twin-domains^2^. Ferroelasticity is not an intrinsic ferroic property that is related to lattice polarization but rather a phenomenon reflecting the strain-stress interplay of material. Hence, the fundamental question about the relation between ferroelasticity and ferroic properties in MHP materials should be addressed.

In this work, the ferroelastic MAPbI_x_Cl_3-x_ twinning-tetragonal phase (tT-phase) was fabricated by stimuli-triggered symmetry breakdown. It was found that the non-radiative recombination in tT-phase MAPbI_x_Cl_3-x_ can be significantly suppressed under electric activation, and the *V*_oc_ of the solar cells is remarkably boosted. Moreover, microscopic and macroscopic characterization results self-consistently revealed the synergistic effect of ferroelasticity on the photovoltaic performances of MHP solar cells. Our work highlights the important role of ferroelasticity in the formation of lattice polarization, which provides a new understanding of charge separation in MHPs.

## Results

This work is organized as follows: the formation mechanism of ferroelastic twin-domains is studied. Then the impact of ferroelastic switching on the non-radiative recombination, as well as device performances, is investigated in detail. Possible mechanisms of the interplay between ferroelastic lattice deformation and lattice polarization are experimentally and theoretically verified.

### Stimuli-triggered symmetry breakdown

The crystallization temperature of MAPbI_x_Cl_3-x_ is carefully controlled to manipulate the symmetry breakdown during post-annealing cooling. The MAPbI_x_Cl_3-x_ film is fabricated on glass/indium tin oxide (ITO)/poly[bis(4-phenyl) (2,4,6-trimethylphenyl) amine (PTAA) by one-step spin-coating, the sample is then annealed at a temperature (${T}_{a}$) for crystallization and subsequently cooled down to a specific temperature of 20 °C (detailed fabrication process can be found in the Methods section).

**Fig. 1:**
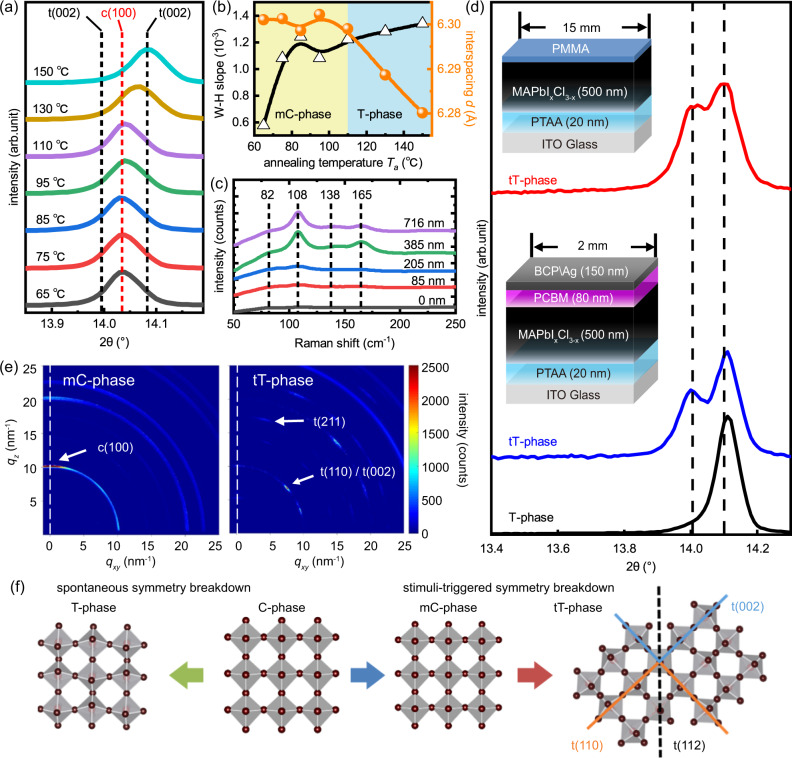
Formation mechanism of the tT-phase. **a** XRD patterns of MAPbI_x_Cl_3-x_ annealed at various temperatures. **b** Interspacing (circle) and W–H slope (triangle) versus annealing temperature (T_a_) of MAPbI_x_Cl_3-x_. **c** Raman spectra of PTAA/MAPbI_x_Cl_3-x_ stack (0 nm) and PTAA/MAPbI_x_Cl_3-x_/PMMA stacks with various PMMA thicknesses. **d** XRD pattern of T-phase and tT-phase MAPbI_x_Cl_3-x_, the schematic layouts are presented as insets. **e** GIWAXS patterns of mC-phase and tT-phase MAPbI_x_Cl_3-x_. **f** a schematic drawing of the crystallographic evolution of stimuli-triggered symmetry breakdown.

Figure 1a presents the X-ray diffraction (XRD) patterns of MAPbI_x_Cl_3-x_ films fabricated at a ${T}_{a}$ varied from 65 to 150 °C (full XRD spectrum is presented in Supplementary Fig. 1). For films fabricated at ${T}_{a}$ > 95 °C, the main diffraction peak (*2θ* ≈ 14°) is systematically shifted toward a higher angle. This diffraction peak can be attributed to either (100) plane-set of C-phase or (110), (002) plane-sets of T-phase^26^, the ascription of which should be considered from a symmetry point of view. The diffraction peak (2θ ≈ 23.5°) belongs to a high order plane-set of (211), which only exists in T-phase^27^. It is observed that the T-phase signature of (211) peak can only be observed from ${T}_{a}$ = 150 °C samples (Supplementary Fig. 1), indicating the spontaneous C- to T-phase transition only occurs at a high ${T}_{a}$. Hence, the diffraction peaks at 14.04 ° and 14.10 ° are assigned to (100) plane-set of C-phase and (110) plane-set of T-phase, respectively^28,29^. To ease the discussion in the following sections, the crystallographic notations are marked with respect to the phases, as t(110) is referred to as the (110) planes of T-phase, and the metastable high-symmetry phase is referred to as the metastable-cubic phase (mC-phase) to distinguish it from the C-phase that exists at a temperature above the phase transition point^30^.

The high-symmetry phase observed at room temperature is formed due to the decrease in internal energy when cooling from a lower ${T}_{a}$ is inadequate to compensate the entropy change of the C- to T-phase transition. Under various ${T}_{a}$ (Supplementary Fig. 1), the shape of the diffraction peak is different, as evaluated by the Williamson–Hall (W–H) relationship^31,32^, it is observed that the microstrain is elevated along with the increase of ${T}_{a}$ (Fig. 1b). The observation is in accordance with the trend of decrease of internal energy change lead to elevated entropy^33^, i.e., the promoted the non-uniform lattice deformation of the mC-phase. Moreover, if mC-phase is a high entropy phase, it should be responsive to further change on internal energy. It is known that poly(methyl-methacrylate) (PMMA) interacts with non-coordinated Pb atoms to form PMMA-Pb_X_ adduct and minimize the total Gibbs free energy^34,35^. For comparison, two types of MAPbI_x_Cl_3-x_ (metastable high-symmetry mC-phase and low-symmetry T-phase) are coated with PMMA and the symmetry is investigated by the Raman spectroscopy^36^. As shown in Fig. 1c, the pristine mC-phase MAPbI_x_Cl_3-x_ (PTAA/MAPbI_x_Cl_3-x_ stack) does not show a prominent Raman signal. However, after it was coated with PMMA, Raman signals at 108 and 165 cm^−1^ can be observed, the signals can be attributed to the liberational modes of the interaction between ${{{{{{{\mathrm{MA}}}}}}}}^{+}$ and ${{{{{{{\mathrm{PbI}}}}}}}}_{3}^{-}$ octahedral^37^. Observation of these Raman peaks is an indication of a higher degree of conformational constraint between ${{{{{{{\mathrm{MA}}}}}}}}^{+}$and ${{{{{{{\mathrm{PbI}}}}}}}}_{3}^{-}$ octahedral, which is direct evidence of symmetry breakdown of mC-phase triggered by the external stimuli. The XRD study further confirms stimuli-triggered symmetry breakdown as the emergence of tetragonal-related peaks in mC-phase MAPbI_x_Cl_3-x_ covered with PMMA (Supplementary Fig. 2). In comparison, T-phase MAPbI_x_Cl_3-x_ covered with PMMA did not show changes in symmetricity (Supplementary Fig. 3). It should also mention that the symmetry breakdown of mC-phase MAPbI_x_Cl_3-x_ can also be triggered by multiple approaches, as the twin-peaks can be clearly observed from mC-phase MAPbI_x_Cl_3-x_ in PTAA/MAPbI_x_Cl_3-x_/PCBM/BCP/Ag stack as shown in the inset of Fig. 1d.

Furthermore, it is found that the crystallographic structure of the low symmetry phase obtained from stimuli-triggered symmetry breakdown is different from the counterpart from spontaneous phase transition. As shown in Fig. 1d (the full XRD spectrum is presented in Supplementary Fig. 4), the c(100) diffraction peak that initially at 14.04° is split into t(002)/t(110) twining peaks located at 14.00° and 14.10°, respectively^2,24^. The observation suggests that the stimuli-triggered symmetry breakdown of mC-phase is anisotropic, i.e. the symmetry of c{100} planes become t{002} or t{110} planes along multiple c < 100> directions. As a result, the low symmetry phase is composed of T-phase domains with two orientations and related through a lost symmetry element during symmetry breakdown. An apparent crystallographic texture is associated with the stimuli-triggered symmetry breakdown, as evidenced by the significantly reduced XRD intensity (Supplementary Fig. 4). The crystallographic texture is well-correlated with the observation from the Grazing Incidence Wide-Angle X-ray Scattering (GIWAXS)^38^. As shown in Fig. 1e, a two-dimensional GIWAXS image of mC-phase (PTAA/MAPbI_x_Cl_3-x_ stack) presents a sharp Bragg spot (*q* = 10 nm^−1^) at the out-of-plane direction. On the contrary, the diffraction signal at the out-of-plane direction in PTAA/MAPbI_x_Cl_3-x_/PCBM/BCP/Ag stack reduced significantly, and the Bragg spot (*q* = 10 nm^−1^) is shifted to an azimuth angle of ~45°. The crystallographic texture can be readily observed from Supplementary Fig. 5. More importantly, GIWAXS result confirms the previous prediction that the formation of twin-domains, as evidenced by the existence of two Bragg spots at one azimuth angle (Supplementary Fig. 6). The observation indicates that the stimuli-triggered symmetry breakdown is anisotropic.

A schematic sketch of the phase transition is shown in Fig. 1f, the stimuli-triggered symmetry breakdown is composed of the fabrication of mC-phase (blue arrow) and the external stimuli (red arrow), which is different from the traditional spontaneous symmetry breakdown (green arrow). For ease of discussion in the following, the twinning-tetragonal phase is denoted as tT-phase to distinguish it from the T-phase obtained from spontaneous phase transition. Moreover, the relative population of twin-peaks A_t(002)_/A_t(110)_, where A_(hkl)_ represents the area under the diffraction peak. The relative population (Supplementary Fig. 2), as well as the interspacing (Supplementary Fig. 7), are all well-correlated with the strain level, which is in accordance with the nature of ferroelastic twin-domains^2,20^.

### Suppression of non-radiative recombination in twin-domains

The essential feature of ferroelasticity is strain-stress retention^2,3^. In polar crystals, the ferroelastic lattice deformation may be related to the lattice polarization^39^, which has a critical role in the charge carrier dynamics^39,40^. Herein, the tT-phase MAPbI_x_Cl_3-x_ is adopted as absorbers in inverted perovskite solar cells, the device layout is presented in Fig. 1d and a cross-sectional SEM of the device is presented in Supplementary Fig. 8.

**Fig. 2:**
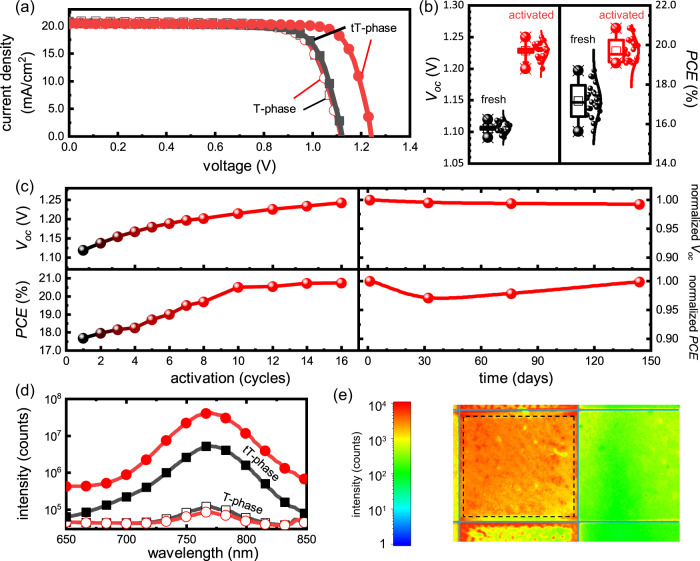
Photovoltaic performance of tT-phase solar cells. **a** J-V curves of T-phase (open marker) and tT-phase (solid marker) MAPbI_x_Cl_3-x_ solar cells before (square) and after (circle) the activation. **b** *V*_oc_ and PCE of fresh and activated tT-phase MAPbI_x_Cl_3-x_ over 23 devices, fitted with normal distribution curves. **c** Activation process and stability of tT-phase MAPbI_x_Cl_3-x_ device. **d** Steady-state PL of T-phase (open marker) and tT-phase (solid marker) MAPbI_x_Cl_3-x_ devices before (square) and after (circle) the activation. **e** PL imaging of the active area of an activated tT-phase solar cell, solid lines mark the positive and negative contacts, and dashed lines mark the active area as it is defined by the overlap of contacts.

Figure 2a presents the current density versus voltage (J-V) curves of tT- and T-phase devices (with the external quantum efficiency of the tT-phase device presented in Supplementary Fig. 9). An enhancement of power conversion efficiency (PCE) under continuous electric activation was observed for tT-phase devices. In contrast, no performance variation was observed for the T-phase devices. The electric activation was controlled by the number of activation cycles (technic details concerning the activation is introduced in the Methods section and J-V curve of one activation cycle is presented in Supplementary Fig. 10). We conducted electric activation on two types of tT-phase solar cells (Supplementary Fig. 10) with a valid area of 0.04 cm^2^ (Supplementary Table 1) and 1.00 cm^2^(Supplementary Table 2), respectively. It is found that the performance of the solar cell is elevated for both cases.

The activation of tT-phase devices is highly reproducible, as displayed in Fig. 2b, the average PCE of 23 devices increased from 17.16 to 19.69% after the activation. The PCE increase mainly originated from *V*_oc_ and fill factor (FF), the continuous electric activation led to a *V*_oc_ increase from 1.12 to 1.26 V and a fill factor increase (FF) from 77 to 81%. As shown in the left part of Fig. 2c, it is found that the progressive increase reached its limit after electric poling for ~16 cycles. Consequently, the PCE of the champion device was increased from 17.91 to 20.93% with negligible hysteresis (Supplementary Fig. 12), the detailed photovoltaic parameters of the champion devices are listed in Table 1.

**Table 1:** Photovoltaic parameters of T-phase and tT-phase MAPbI_x_Cl_3-x_ devices

Sample	Status	*V*_oc_ (V)	*J*_sc_ (mA/cm^2^)	FF (%)	PCE (%)
**T-phase**	fresh	1.07	21.29	72.80	16.59
	activated	1.07	21.24	72.68	16.51
**tT-phase**	fresh	1.10	20.06	76.93	17.91
	activated	1.23	20.05	81.02	20.93

The effect of electric activation is stable, as the photocurrent under fixed bias shows no sign of decrease (Supplementary Fig. 13). To further investigate the recoverability of the activation effect, a long-term stability analysis was carried out on the fully activated solar cells. As demonstrated in the right part of Fig. 2c, no signs of deactivation or degradation were observed during the stability test for over 150 days. The observation indicates that the *PCE* increase induced by electric activation is everlasting.

The elevated *V*_oc_ is an indication of an increase in quasi-Fermi level splitting. And according to the optoelectronic reciprocity relationship^41,42^, the lowest *V*_oc_ loss corresponds to the minimum non-radiative recombination and thereafter, the highest radiative recombination. In this regard, steady-state photoluminescence (PL) spectroscopy was adopted to probe the radiative recombination of MAPbI_x_Cl_3-x_ in a complete device. As shown in Fig. 2d, the PL intensity of the tT-phase device was enhanced by more than one order of magnitude after the activation. On the contrary, negligible PL change was observed for the T-phase device after equivalent activation. In order to present a complete picture, PL imaging was performed on the active area of the solar cell (Supplementary Fig. 14). Figure 2e illustrates the PL image of a tT-phase device after activation, where the active area is defined by the overlap of the front and back electrodes (as marked by dotted lines). An apparent PL contrast was observed between the active and inactive area of the tT-phase device, indicating that the non-radiative recombination in the active area is uniformly lower than that in the inactive area, while such contrast is absent from that of T-phase devices (Supplementary Fig. 15). The presence of an especially bright area at the edge of an active area of the tT-phase devices is probably caused by the non-uniform electric field distribution, such effect has been reported in the optoelectronic devices with similar structure^43^. The significant enhancement of PL intensity from the active area is surprising as the electrodes usually suffer additional surface recombination, which commonly reduces the PL intensity^44,45^. On top of that, transient PL measurement indicates that the averaged carrier lifetime is increased after the activation (Supplementary Fig. 16), along with the increased PL intensity (Fig. 2d), our results indicate that the non-radiative recombination is apparently suppressed after the activation.

### Elevated photovoltaic performance by the synergistic effect of ferroelasticity

**Fig. 3:**
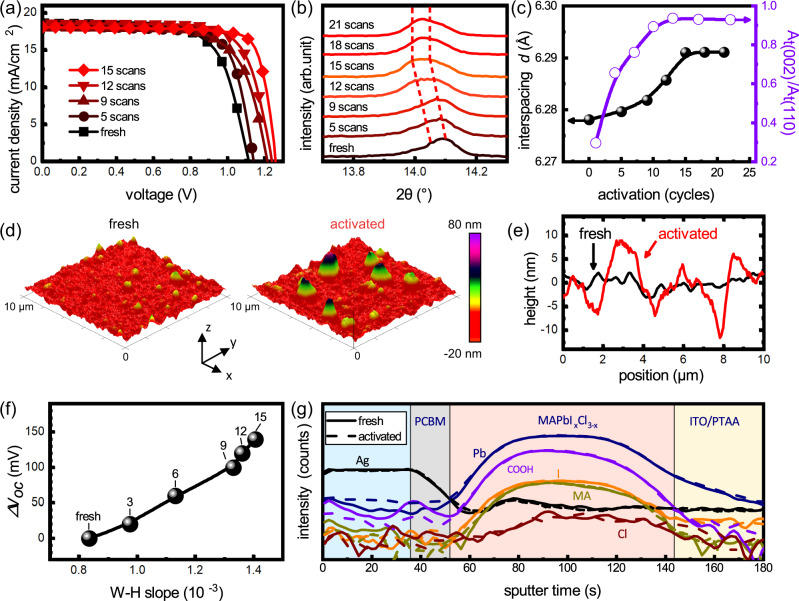
Ferroelastic deformation of tT-phase under electric activation. **a** J-V and **b** XRD of tT-phase MAPbI_x_Cl_3-x_ solar cells activated to different levels (from 1.12 V to 1.26 V). **c** interspacing of t(110) plane-set *d* (solid) and relative population of twin-domains A_t(110)_/A_t(002)_ (open) versus activation cycles. **d** topography of fresh and activated tT-phase device. **e** Averaged height versus position on *x* direction. **f** The enhancement of open-circuit voltage*△V*_*oc*_ versus the W–H slope, labels indicate the activation cycles. **g** Depth profiling element concentration of fresh and activated tT-phase solar cells.

In order to understand the suppression of non-radiative recombination under electric activation, the crystallographic structure of tT-phase MAPbI_x_Cl_3-x_ was investigated along with the activation process. As shown in Fig. 3a, the tT-phase devices were activated to different levels by manipulating the repetition of activation cycles. The XRD patterns of tT-phase devices along with the activation cycles were measured (Fig. 3b, with full spectrum presented in Supplementary Fig. 17), it is observed that the diffraction peaks of t(110) and t(002) plane-sets were progressively shifted toward a lower angle along with the activation process. It should be noted that such deformation is observed after the withdrawal of the electric field, indicating the crystallographic deformation induced by an electric field is self-stabilizable. Moreover, the observation suggests the degree of lattice deformation can be modulated by the repetition cycles. Furthermore, to have a more clear picture of the crystallographic structure, the XRD of tT-phase devices were fitted with the Voigt function to deconvolute t(110) and t(002) signals, and the interspacing ${d}_{t(110)}$ and relative population of twin-domains ${{{{{{\rm{A}}}}}}}_{{{{{{\rm{t}}}}}}(002)}/{{{{{{\rm{A}}}}}}}_{{{{{{\rm{t}}}}}}(110)}$ were calculated and plotted versus the counts of the activation cycles, the results are shown in Fig. 3c (detailed data is listed Supplementary Table 4). It is observed that the interspacing and the relative population of twin-domains were elevated progressively along with the activation progresses. In contrast, no persistent crystallographic evolution can be observed from T-phase devices (Supplementary Fig. 18). The observation suggests that the self-stabilizable crystallographic deformation is associated with the switching of ferroelastic domains.

A topographic variation could be induced by irreversible crystallographic deformation under electric activation. It is known that the morphology is connected to the strain level^46^ and the morphology of MHP materials could be recoverably changed under electric poling due to the electrostriction effect^47^. However, this is not the case for the ferroelastic twin-domains, as a permanent topography change was detected. Atomic force microscopy (AFM) was adopted to study the surface morphology. As shown in Fig. 3d, AFM topography images of tT-phase devices before and after electric activation are presented in parallel. The topography of the fresh tT-phase device is smooth and the morphology of the crystal grains can be clearly observed. In contrast, the surface roughness of the activated device is remarkably increased, with grains apparently elongated along the out-of-plane direction. The averaged surface height of the devices is presented in Fig. 3e, where the morphology variation can be readily observed.

## Discussions

Up mentioned results presented a comprehensive picture of crystallographic deformation along with electric activation. The irreversible change of surface morphology is in good agreement with the self-stabilizable crystallographic deformation under an electric field. The discrepancy in recoverability between this work and the earlier literature report^47^ is due to the presence of ferroelastic twin-domains. Under an electric field, electrostriction elevates the strain level in unit-cell so that the orientation of PbI_3_^−^ octahedral at domain walls may be switched to become more energetically favorable, thus leading to the shift of the domain walls^2,24,48^. Similar to the twin-domains in inorganic perovskites, the domain walls tend to be pinned down by the attraction of the X-site vacancies under strain variation^49^, so that domain wall as well as interspacing, cannot shift back to the original position after the withdrawal of electric field, the irrecoverable shifting of domain wall establishes the self-stabilizable lattice deformation under electric activation. More importantly, we found the strain level is elevated along with the electric activation (Supplementary Fig. 19), this is reasonable as the strain level is regulated by the ferroelastic strain-stress retention and the interspacing is self-stabilizable increased by ~2‰ during the electric activation. As shown in Fig. 3f, the elevated strain level is in good correlation with the *V*_oc_ increment of as much as 150 mV.

In literature, enhancement of the photovoltaic performance under illumination or electric field is usually interpreted as light soaking^50^, ion migration^51–53^, and modulation of lattice polarization^12,40^. For example, Tsai et al. found that the photovoltaic performance of MHP-based solar cells could be enhanced by illumination-induced uniform lattice expansion, which consequently reduced both the energy barrier at interfaces and non-radiative recombination in the bulk^50^. However, multiple observations of electric activation are not aligned with this mechanism. Firstly, the activation performances of our solar cell lasted at least for months, which contradicts the general observation of recoverable lattice expansion caused by light soaking. Secondly, elevated strain instead of relaxed strain was observed in the activated device. The W–H slope is apparently increased after the electric activation (Supplementary Fig. 19), indicating an elevated strain level after activation. Thirdly, the activation effect is independent of light illumination. As evidenced by the control experiments of the intentionally short-circuited devices cannot be activated, on the contrary, the device can be activated by an external electric field in the dark (Supplementary Fig. 20). The observations establish the fact that it is the electric field triggers the activation effect instead of illumination.

Another possible explanation for electric activation is ion migration. MHP materials are known to have ion migration behavior under an electric field^52,53^. The accumulation of ions may lead to uneven doping and thereafter enhance charge separation^54^. The ion migration behavior in MHP materials has been extensively studied^55^ and two types of ions are reported to have favorable migration under an electric field: fast mobile halide ions (I^−^, Cl^−^) with an activation energy of ~0.3 eV and slow mobile ions of MA^+^ cations (~1 eV)^56,57^. Secondary ion mass spectrometry (SIMS) can provide depth profiling of ion concentrations. As shown in Figure 3g, SIMS was adopted to investigate the redistribution of the abovementioned ions (MA^+^, I^−^, Cl^−^) in tT-phase MAPbI_x_Cl_3-x_ before and after activation. It is found that the depth profiles of ion concentrations for the devices before and after the activation are almost overlapped, indicating that ion redistribution cannot afford the enhancement of charge separation hereby observed.

Then, let’s focus on the lattice polarization. Several recent studies suggest the existence of lattice polarization in MHP under stain^40,58,59^. Hence, it is reasonable to assume that the activation triggers the formation of lattice polarization as the self-sustainably elevated strain level is in good correlation with the *V*_oc_ increment (Fig. 3f). We performed first-principles calculations on the tetragonal MHP under different strains considering the ferroelasticity of tT-phase MHP. The MA^+^ molecule is replaced with a single Cs^+^ atom, which has an effective size similar to that of the MA^+^ molecule, i.e., we study the polarization properties of CsPbI_3_.

**Fig. 4:**
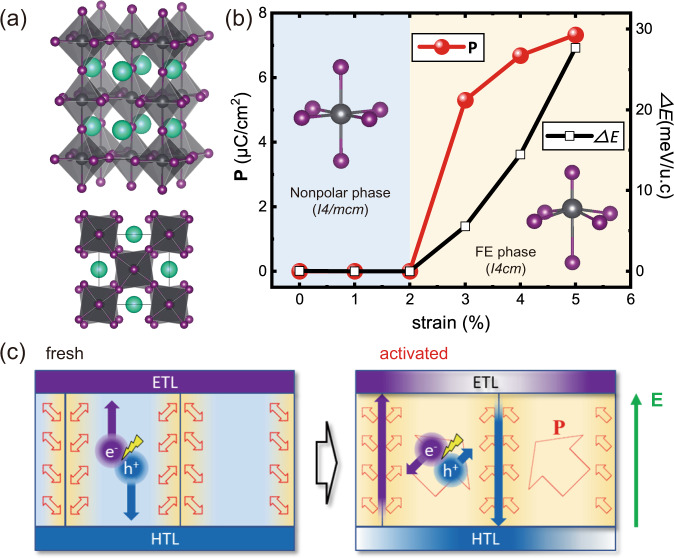
Synergistic effect of ferroelasticity and photovoltaic. **a** Crystal structure of tetragonal CsPbI_3_ in our first-principles simulations. (**b**) Structural phase transition and ferroelectric polarization induced by strain. The nonpolar phase has a space group of *I4/mcm*, while the ferroelectric phase has a space group of *I4cm*. **c** Schematic illustration of the effect of lattice polarization on suppressing non-radiative recombination in electrically activated MAPbI_x_Cl_3-x_ twin-domains. For twin-domains of fresh tT-phase, spontaneous lattice polarization only occurs at the domain wall, but there is no long-range ferroelectric order (double-sided arrows). For the activated tT-phase MHP, the external electric field (**E**) aligns all polarizations at the domain wall and further induces ferroelectric polarization (**P**) inside the domains (hollow arrows) and photoexcited electrons and holes moving towards the opposite domain wall.

As shown in Fig. 4a, CsPbI_3_ has the same space group as MAPbI_3_, i.e., *I4/mcm* or *I4cm*, depending on whether PbI_3_ has a ferroelectric distortion (relative position shift between Pb and I) besides the same octahedral rotation. The optimal lattice constants of CsPbI_3_ are *a* = *b* = 8.84 Å, *c* = 12.94 Å, matching well with the experimental values of MAPbI_3_ (*a* = *b* = 8.86 Å, *c* = 12.64 Å). The calculated polarization of CsPbI_3_ as a function of strain is shown in Fig. 4b. Without strain, tetragonal CsPbI_3_ is non-polarized and has a centrosymmetric *I4/mcm* structure. When the tensile strain is larger than 2%, a structural phase transition occurs from non-polarized *I4/mcm* to ferroelectrically polarized *I4cm*. The polarization is mainly induced by the relative shifts of Pb and I atoms along the *c* direction, as shown in the inset of Fig. 4b.

The above-calculated results conclude that the T-phase MAPbI_x_Cl_3-x_ has no polarization before and after electric activation. Therefore, there should be no change in non-radiative recombination for both fresh and activated T-phase MAPbI_x_Cl_3-x_, as demonstrated by no photovoltaic performance variation in our experiments. For the tT-phase MAPbI_x_Cl_3-x_, ferroelastic, non-uniform strains are distributed around the frequently existing domain walls^46,47^. Hence, spontaneous polarization should occur near the domain wall in the tT-phase, consistent with the recently calculated results^46,47^. However, no long-range ferroelectric order is formed across the domain and domain wall due to the release of strain inside the domains, as schematically shown at the left of Fig. 4c.

Under the activation of an electric field, all the polarizations are aligned in the same direction in a domain. At the same time, ferroelectric polarization is also induced inside the domain. Considering the applied electric field on MAPbI_x_Cl_3-x_ is equivalent to the *V*_oc_ of devices, which is 1.1 V at minimum (*V*_oc_ of a fresh device) and the thickness of absorber is ~500 nm at maximum, then the applied electric field is 22 kV/cm at minimum, which is large enough to align or reverse the polarization direction at the domain walls, as demonstrated in the recent works^60,61^. Due to the ferroelasticity, the activation will irreversibly deform the crystallographic structure of domain walls, as revealed by our ${{{{{{\rm{A}}}}}}}_{{{{{{\rm{t}}}}}}(002)}/{{{{{{\rm{A}}}}}}}_{{{{{{\rm{t}}}}}}(110)}$ and interspacing measurements in Fig. 3c. Hence, such ferroelectric polarization is maintained even after the withdrawal of the external electric field. Considering the direction of the domain wall between t(002) and t(110) domains, the actual polarization has an angle of 45° to the domain wall.

The synergistic effect of lattice polarization and photovoltaic electric field drives the separation of photoexcited charge carriers and the coupling of the electric fields is similar to that in a gate-controlled transistor^43^. Under illumination, The lattice polarization maintained by ferroelastic lattice deformation will facilitate charge separation at an atomistic scale. Photoexcited electrons and holes will be injected into opposite domain walls (right of Fig. 4c), where the population of positive and negative carriers will be built-up. Then the carriers will be injected into the anode and cathode under the photovoltaic electric field. And the non-radiative recombination is remarkably suppressed due to the diffusion of carriers is unimpeded by the carriers with opposite charges under such a synergistic effect.

In summary, our work highlights the important role of ferroelasticity in the photoelectronic properties of MHPs. We revealed the formation of MAPbI_x_Cl_3-x_ twin-domains via the method of stimuli-triggered symmetry breakdown. The twin-domains are ferroelastic in nature, as characterization results at micro and macro scale self-consistently revealed the irreversible crystallographic deformation under an electric field. It is also found that the non-radiative recombination of twin-domains can be significantly suppressed by electric activation. The well correlation between ferroelastic lattice deformation and self-stabilizable suppressed non-radiative recombination indicates the synergistic effect of ferroelasticity is the origin of suppressed non-radiative recombination. Simulation results further revealed the mechanism of a synergistic effect of ferroelasticity, that is, the existence of lattice polarization due to non-uniform strain near the domain wall of twin-domains, the external electric field aligns them and further induces a ferroelectric polarization across the entire domain. The polarization can be maintained due to the ferroelasticity, as the lattice structure is self-ostabilizable after the withdrawal of the electric field. The lattice polarization facilitates charge separation at an atomistic scale and suppresses non-radiative recombination of tT-phase MAPbI_x_Cl_3-x_. Consequently, the *V*_oc_ of tT-phase solar cells can be remarkably enhanced to 1.26 V, which is approaching the theoretical limit of the material. This work also highlights the important role of post-treatment in studying the lattice-related properties of MHPs. Due to the nature of flexible lattice structure, it is challenging to precisely manipulate the lattice structure of MHPs. As a post-treatment, electric activation naturally excludes the undesired impacts from fabrications. As a matter of fact, post-treatments have drawn more attention in recent works to study the lattice-related properties of MHPs^62,63^. The synergistic effect enabled a method to understand the origin of low non-radiative recombination of MHP materials. The observations hereby reported reveal the important role of ferroelastic stress on the charge separation of MHP materials, those results provide new and critical understandings of the excellent performance of MHP solar cells.

## Methods

### Materials

Methylammonium iodide (MAI) was purchased from Great Cell Solar. Lead acetate trihydrate (Pb(CH_3_COOH)_2_ ∙ 3H_2_O, >99.5%) was purchased from TCI. Lead chloride (PbCl_2_, >99.999%) was purchased from Alfa Aesar. Poly [bis (4-phenyl) (2,4,6-trimethylphenyl) amine] (PTAA) and Bathocuproine (BCP) were purchased from Xi’an p-OLED Corp. [6,6]-phenyl-C_61_-butyric acid methyl ester (PCBM) was purchased from Solarmer. Polymethyl methacrylate (PMMA) was purchased from Shanghai Macklin Biochemical. Toluene, dimethylformamide (DMF), isopropanol (IPA), ethanol, and chlorobenzene (CB) were purchased from Sigma Aldrich and used as received.

### Preparation of mC-phase, tT-phase, and T-phase MAPbI_x_Cl_3-x_

The patterned (1.5 cm^2^ × 1.5 cm^2^) indium doped tin oxide (ITO) glass were purchased from Advanced Election Technology co.Ltd. ITO glasses were ultrasonically washed with IPA and ethanol each for 10 min. The washed ITO substrates were treated with oxygen plasma for 20 min and then transferred to the N_2_-filled glovebox. The hole transport layer of PTAA was fabricated by spin-coating. Particularly, 50 μL of PTAA solution (2.0 mg/mL in CB) solution was spin-coated on ITO glass at 4500 rpm for 20 s (with a ramping rate of 4500 rpm/s), the as-prepared PTAA films were annealed at 110 °C for 10 min. The PMMA films were fabricated by spin-coating, 80 μL precursor solution (25–100 mg/mL in CB, the thickness versus solution concentration plot is presented in Supplementary Fig. 4) was spin-coated at 1000 rpm for 60 s (with a ramping rate of 1000 rpm/s). MAPbI_x_Cl_3-x_ films were fabricated by a two-step spin-coating process. The precursor solution of MAPbI_x_Cl_3-x_ (0.57 M) was prepared by mixing Pb(CH_3_COO)_2_·3H_2_O (0.52 M), PbCl_2_ (0.047 M), and MAI (1.71 M) in 1 mL of DMF. The as-prepared solution was stirred overnight and filtered with a 0.45 µm PTFE filter before use. About 80 μL precursor solution was dropped on the surface of PTAA, consecutive program at 1200 rpm for 10 s (with a ramping rate of 1200 rpm/s) and 6000 rpm for 30 s (with a ramping rate of 1500 rpm/s). The precursor films were annealed for 2 min on a hot plate for crystallization. For the annealing temperature is 75 and 110 °C for the mC-phase and T-phase samples, respectively. After the annealing, the samples are cooled to a specific temperature of 20 °C to avoid undesired spontaneous phase transition. The MAPbI_x_Cl_3-x_ films that were used for characterization were fabricated by using 0.57 M solution.

### Device fabrication

T-phase and tT-phase MAPbI_x_Cl_3-x_ were adopted as absorbers in the inverted solar cells. The solar cell has a layout of Glass/ITO/PTAA/MAPbI_x_Cl_3-x_/PCBM/BCP/Ag. The concentration of MAPbI_x_Cl_3-x_ solution was 0.66 M. Precursor solution of MAPbI_x_Cl_3-x_ was prepared by mixing Pb(CH_3_COO)_2_·3H_2_O (0.60 M), PbCl_2_ (0.059 M), and MAI (1.89 M) precursors in DMF, the as-prepared solution was stirred overnight and filtered with a 0.45 µm PTFE filter before use. The MAPbI_x_Cl_3-x_ film was fabricated on top of ITO/PTAA by using the same spin-coating program as mentioned above. The electron transport layer of PCBM was fabricated as follows: 50 μL PCBM solution (20 mg/mL in chlorobenzene and toluene) was spin-coated on the top of MAPbI_x_Cl_3-x_ layer at 1000 rpm for 30 s. About 60 μL BCP (0.5 mg/mL in IPA) was spin-coated at 4000 rpm for 20 s. At last, 80 nm Ag was deposited with a shadow mask by thermal evaporation.

### Device performance measurement

The J-V measurement was carried out by using a series 2400 source measure unit (Keithley Instruments), with a scan speed of 0.1 V/s. The illumination was provided by the solar simulator (SS-F5-3A, Enlitech, AM 1.5 G), of which the power density of illumination was 100 mW cm^−2^ as calibrated by a certified standard silicon solar cell (SRC-2020, Enlitech). The valid area of the solar cell is defined by the overlap of the front contact of ITO and the back contact of silver. Each (1.5 cm^2^ × 1.5 cm^2^) sample consists of either four active devices with a valid area of 0.04 cm^2^ or 1 active device with a valid area of 1.0 cm^2^. All the measurements related to the device efficiency were carried out in an N_2_-filled glovebox and an electric fan was used for cooling during the measurement. The devices are stored in a glovebox without encapsulation for stability monitor.

### Electric activation process

The activation process is carried out as repetitive cycles. In this study, there are three types of activation methods. Regular: for each cycle, the device is illuminated at open-circuit status for 180 s. Illumination: for each cycle, the device is illuminated at short-circuit status for 180 s. Electric field: for each cycle, the device is poled at +1.0 V in the dark for 30 s. The illumination is provided by the solar simulator at a power density of 100 mW cm^−2^.

### Crystallographic characterizations

X-ray diffractogram (XRD) is measured by the Rigaku Smart Lab system at room temperature and operated at a current of ~40 mA and voltage of ~45 kV. The Source of the X-ray was Cu with a characteristic wavelength (*λ*) of Cu-Kα equal to 1.54 Å. The diffractogram was measured from 10 to 60°, with a scanning rate of 10°/min.

Grazing incidences of wide-angle X-ray scattering (GIWAXS) is measured at beamline BL14B1 of Shanghai Synchrotron S2 Radiation Facility (SSRF) with the incident photon energy of 10 keV (*λ* = 1.24 Å) at an incident angle of 0.5° and an exposure time of 30 s.

Raman spectrums of mC-phase and tT-phase were obtained by SENTERRA II Raman Microscope. The 532 nm line of a laser was used as an excitation source with a maximum power of 10 mW.

### Photoluminescence-scanned confocal microscopy

We used a home-built photoluminescence (PL)-scanned imaging microscopy coupled with a time-correlated single photon counting (TCSPC) to map the PL dynamics from MAPbI_x_Cl_3-x_ solar cells. Excitation of the sample is achieved with a super continuum white-light laser (SC400-PP, Fianium, UK) of 450 nm wavelength, 2 MHz repetition rate, and ~6 ps pulse width, the power of the laser is calibrated as 1.877 μW. The excitation laser beam focuses on the sample through a 4×objective lens with a spot radius of 1.72 μm. By parking the excitation laser spot in a specific position of the sample, fast scanning is enabled by a galvanometer mirror. Each scanning image contains 256 × 256 pixels with a dwell time of ~1 ms at each pixel. The fluorescence signal is collected using a high-speed detector (HPM-100-50, Hamamatsu, Japan) with a 500 nm long pass filter.

### AFM measurements

The AFM measurements were conducted under a nitrogen atmosphere on Bruker Dimension Icon setup. The topography image is taken from tT-phase solar cells (glass\ITO\PTAA\MAPbI_x_Cl_3-x_\PCBM\BCP\Ag stack), before the topography measurement, the silver electrode is removed by scotch tape and the ETL of PCBM is exposed. For fresh devices, the as-prepared tT-phase solar cell is taken. For the activated device, the tT-phase solar cell is activated until the *V*_oc_ reaches its maximum.

### Williamson–Hall (W–H) analysis

The strain level of the MAPbI_x_Cl_3-x_ devices and films were calculated from Full width and half maximum (FWHM) and peak position (2θ value) by using Eq. (1) ^31,32,64^.

1 $$\beta \cos \theta=\frac{k\times \lambda }{D}+4\varepsilon \times \sin \theta$$

where *β* is the FWHM, *k* is a constant, *λ* is the wavelength of the irradiation, *D* represents the diameter of the particle, and *ε* is the lattice strain. Hence, the relation between *β*×cos*θ* and 4×sin*θ* should give a linear line with the slope of strain (*ε*). The W–H slope was calculated from (002), (110), (202), (220), and (213) planes-sets of the XRD pattern.

### First-principles calculations

The first-principles calculations are performed using the Vienna ab initio simulation package^65^ within the projector augmented wave method^66^ and the generalized gradient approximation of the Perdew–Burke–Ernzerhof^67^ exchange-correlation functional. The plane-wave basis with an energy cutoff of $400\,{{{{{{\mathrm{eV}}}}}}}$ and the Г-centered 7 × 7 × 5 k-point meshes are adopted. To exclude the polarization of the MA^+^ molecule, which is dynamically orientational disordered and doesn’t contribute to ferroelectricity at room temperature, we replace the MA molecule with a single Cs atom. The tetragonal crystal structure of CsPbI_3_ is fully relaxed until the residual forces on each atom is less than 0.01 eV/Å. Nonpolar structures with space group of $I4/{mcm}$ and ferroelectric (space group: $I4{cm}$) are considered. The spin–orbital coupling effect is also taken into account.

### Reporting summary

Further information on research design is available in the Nature Portfolio Reporting Summary linked to this article.

## Supplementary information

Supplementary Information

Solar Cells Reporting Summary

## Data Availability

Data sets generated during and/or analyzed during the current study are available from the first authors and corresponding authors on request. Source data are provided with this paper.

## Source data

Source Data
