# Supplementary material for: Suppressing non-radiative recombination in metal halide perovskite solar cells by synergistic effect of ferroelasticity
Source: Nat Commun. 2023 Jan 17;14:256. doi: 10.1038/s41467-023-35837-1 (PMC9845300; doi:10.1038/s41467-023-35837-1)
Supplement: Supplementary file 1 — Supplementary Information [file 41467_2023_35837_MOESM1_ESM.pdf]

Supplementary Information for

Suppressing Non-radiative Recombination in Metal Halide Perovskite Solar Cells by Synergistic Effect of Ferroelasticity

Wei Qin, Wajid Ali, et al., Can Li

Supplementary Figures

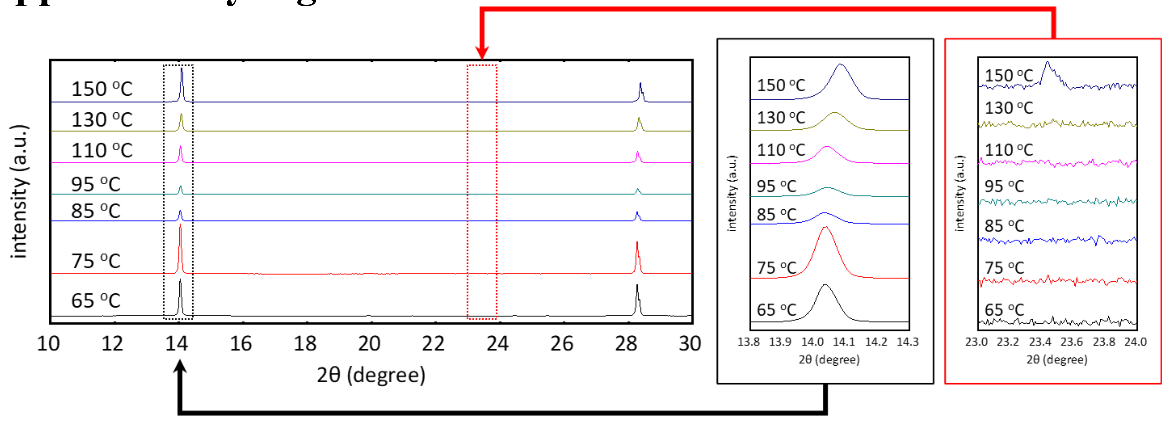

Supplementary Fig.1: XRD full spectrum of $MAPbI_xCl_{3-x}$ films annealed at different temperatures

Supplementary Fig.1 represents the XRD patterns of PTAA/$MAPbI_xCl_{3-x}$ stacks annealed at various temperatures (65-150 °C), the diffraction peaks locate at ~14° belong to t(110) or c(100) plane-set, the diffraction peak locates at ~23.5° belong to t(211) plane-set.

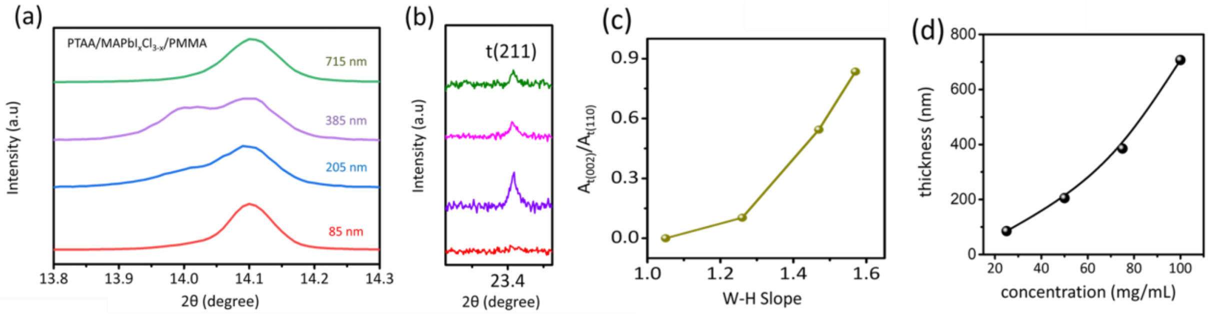

Supplementary Fig.2: XRD pattern of $MAPbI_xCl_{3-x}$(mC-phase)/PMMA stack with various PMMA thicknesses; (a) main diffraction peak, (b) signature diffraction peak of T-phase, (c) relative population of twin-domains versus strain level of $MAPbI_xCl_{3-x}$ films, (d) concentration solution versus the thicknesses of PMMA layer.

Supplementary Fig.2 depicted the XRD pattern $MAPbI_xCl_{3-x}$(mC-phase)/PMMA stack with various PMMA thicknesses. The results revealed that the t(211) tetragonal signature peak could be observed after the $MAPbI_xCl_{3-x}$ mC-phase was covered with 85 nm PMMA layer (Supplementary Fig.2b), which triggered the symmetry breakdown of $MAPbI_xCl_{3-x}$ mC-phase. Further, by increasing the PMMA thickness, the orientation of t(002) and t(110) domains are changing (Supplementary Fig.2c), which confirms the ferroelasticity of $MAPbI_xCl_{3-x}$ under external stress.

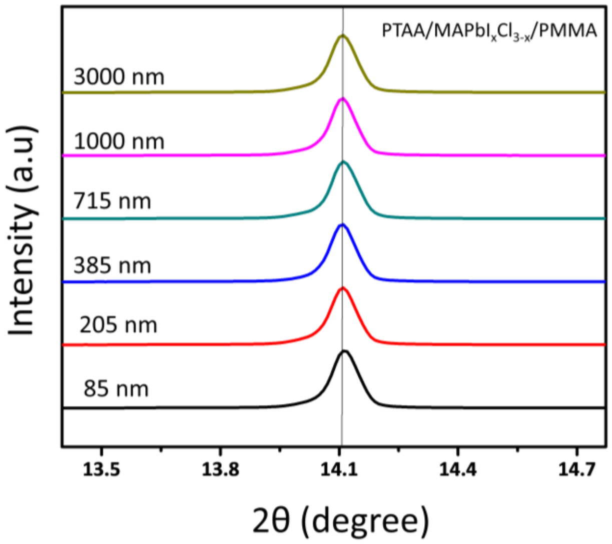

Supplementary Fig.3: XRD pattern of $MAPbI_xCl_{3-x}$(T-phase)/PMMA stack with various PMMA thicknesses.

From the results it is clear that there is no notable difference in the peak position upon deposition of different thickness of PMMA. Hence the results clearly represent that the T-phase $MAPbI_xCl_{3-x}$ would not response to the external stress.

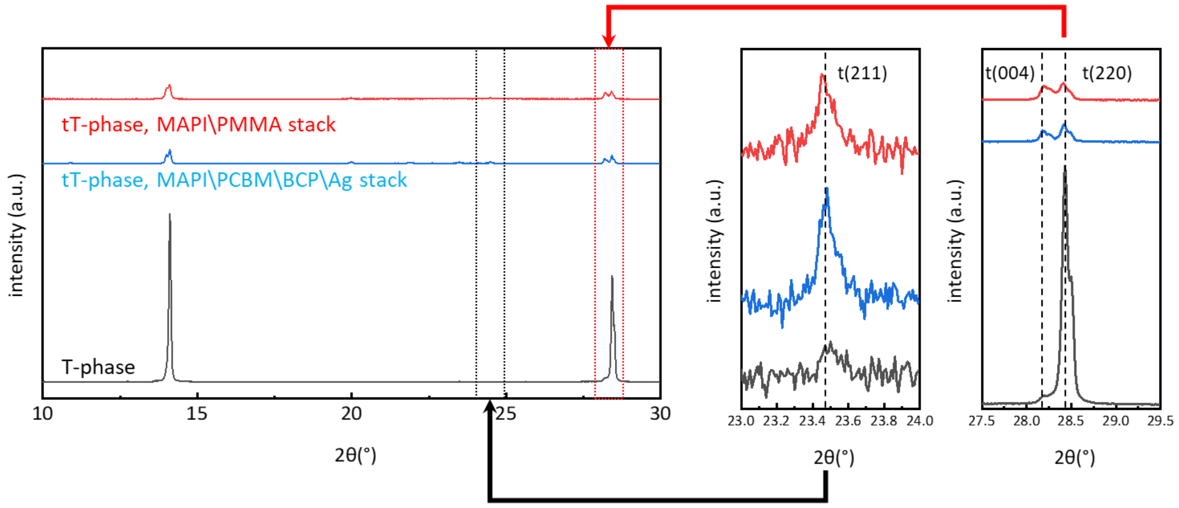

Supplementary Fig.4: XRD spectrum of T-phase and tT-phase $MAPbI_xCl_{3-x}$. The full X-ray diffractogram of mC-phase and tT-phase $MAPbI_xCl_{3-x}$.

It is observed that the tT-phase has a much lower intensity than that of mC-phase, the observation suggests that the stimuli-triggered symmetry breakdown is associated with a crystallographic texture.

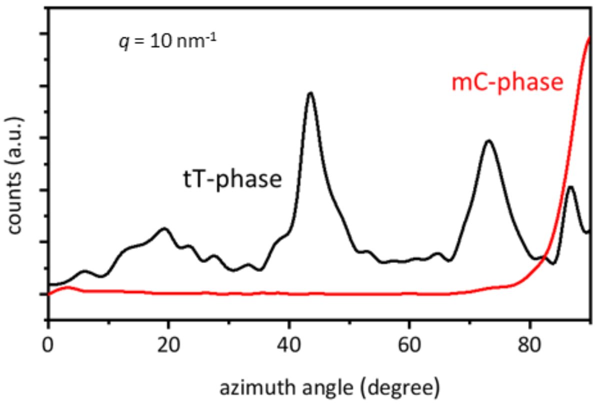

Supplementary Fig.5: diffraction intensity plotted versus azimuth angle

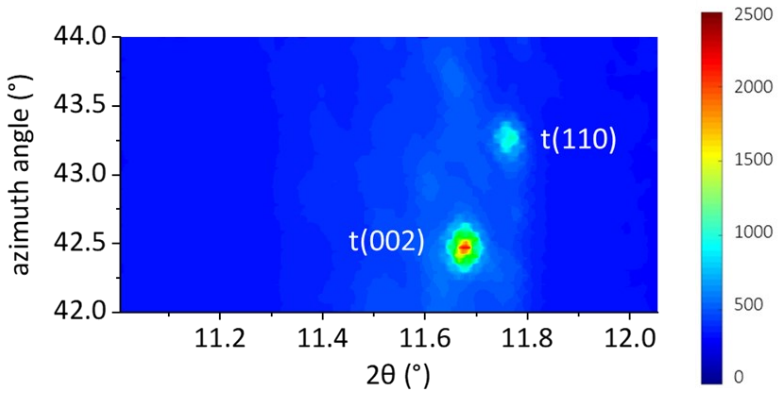

Supplementary Fig.6: diffraction intensity plotted versus azimuth angle and 2theta

As shown in Supplementary Fig.6, it is observed that a small deviation of azimuth angle exists between the Bragg spots, the deviation angle of the twinning plane-sets is caused by the mismatched interspacing of t(110) and t(002) plane-sets, which can be calculated by the following formular[1]:

$$\Delta\omega = \pi - tan^{-1}(d_{t(110)}/d_{t(002)})$$

The calculated separation angle is ~1°, which is in excellent agreement with the observation from Supplementary Fig.6.

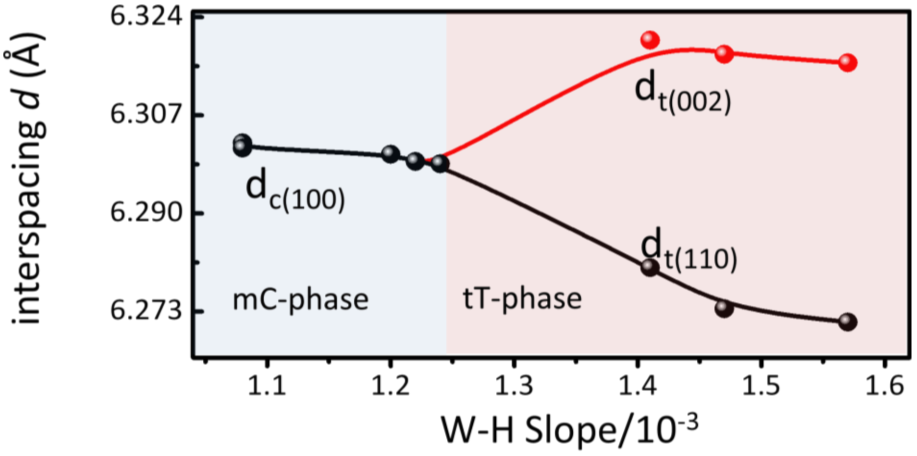

Supplementary Fig.7: interspacing of main diffraction peak (2θ = 14°) versus W-H slope

Supplementary Fig.7 shows the *d* versus W-H slope, as the strain level enhances the $MAPbI_xCl_{3-x}$ change the phase from mC-phase to tT-phase. At particular value of strain the $d_{c(100)}$ convert to $d_{t(110)}$ and $d_{t(002)}$. Here the low symmetry phase obtained from stimuli-triggered symmetry breakdown is different from the counterpart of spontaneous phase transition. It indicated that $MAPbI_xCl_{3-x}$ has composed of T-phase domains with two orientations and related through a lost symmetry element during symmetry breakdown. This is direct evidence of the formation of twin-domains by the stimuli-triggered symmetry breakdown[2,3].

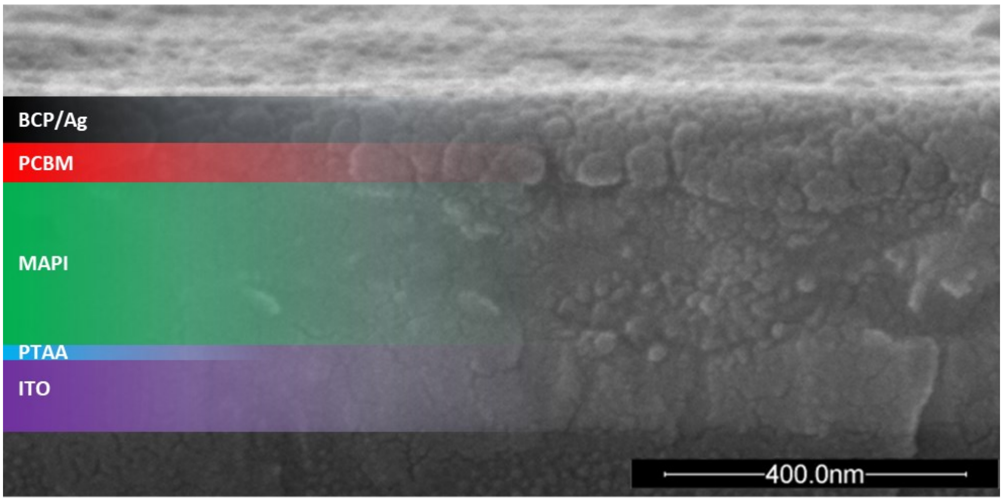

Supplementary Fig.8: Cross-sectional SEM image of the inverted solar cell.

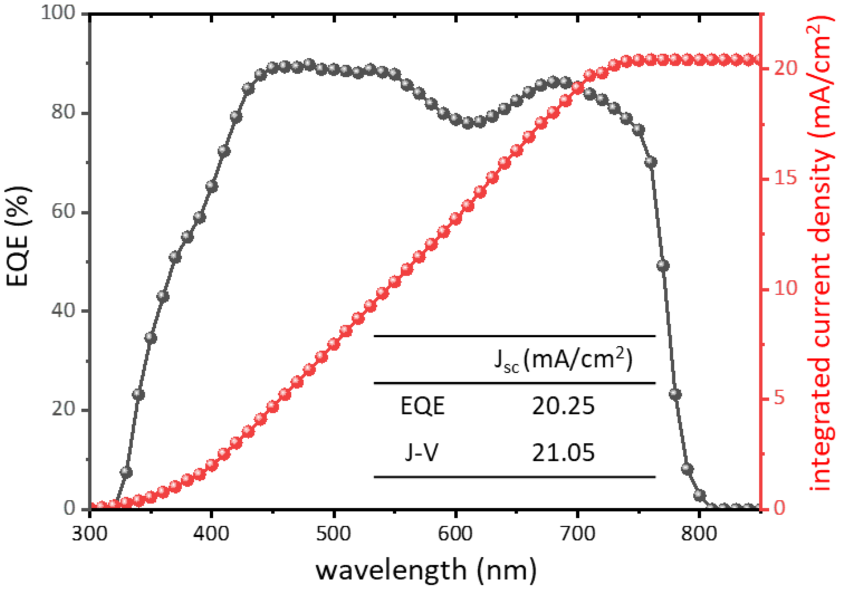

Supplementary Fig.9: external quantum efficiency (EQE) and integrated J of the tT-phase solar cell

The result of EQE measurement is presented in Supplementary Fig.9, it is found that the integrated current density obtained from EQE is comparable with the short circuit current density that obtained from JV scan (Figure 2a).

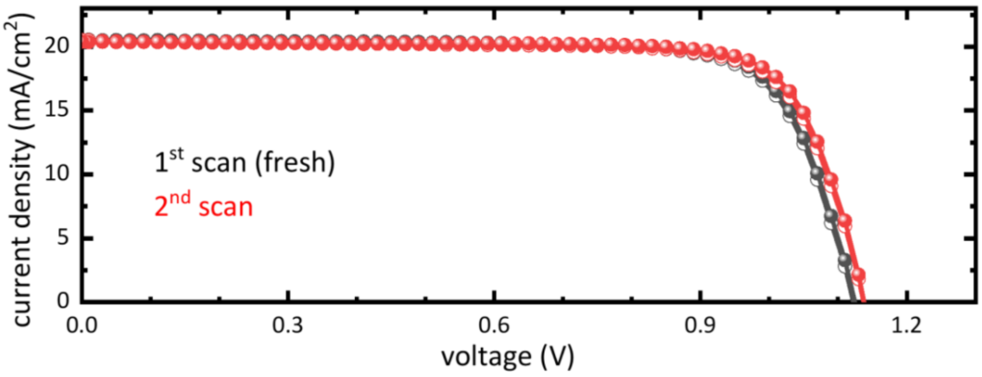

Supplementary Fig.10: current density versus voltage curves of the first and second scan from tT-phase device (solid and open markers indicate reverse and forward scan, respectively).

Increased photovoltaic performance that caused by activation effect, the first J-V scan is shown as black lines in Supplementary Fig.10. The solar cell is then activated for 1 cycle (regular method), an increment on V_{oc} can be readily observed from the 2nd J-V scan (red lines in Supplementary Fig.10).

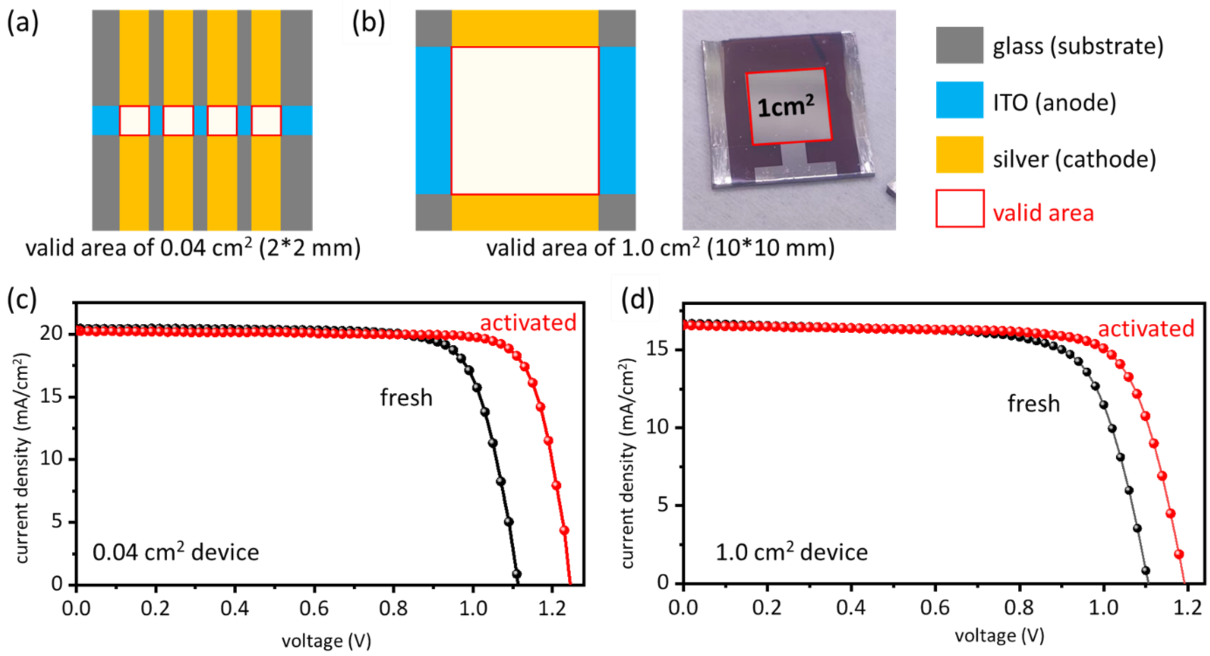

Supplementary Fig.11: (a, b) schematic layout and (c,d) J-V curves of 0.04 cm^2 and 1 cm^2 devices

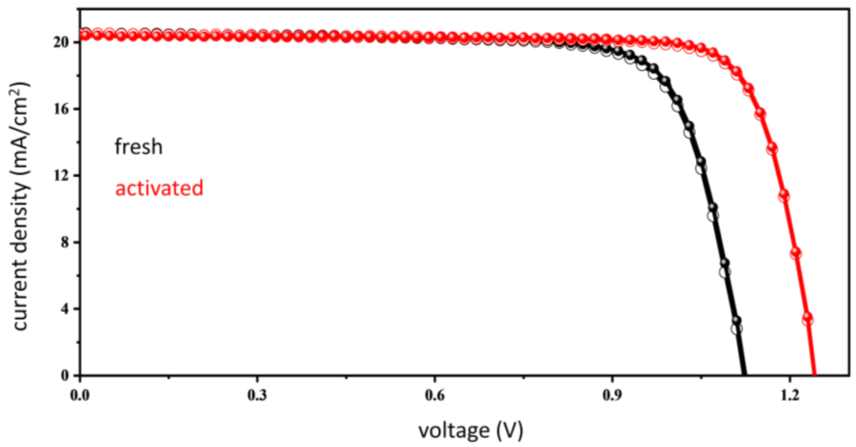

Supplementary Fig.12: current density versus voltage curves of tT-phase solar cell before and after electric activation shows negligible hysteresis (solid and open markers indicate reverse and forward scan, respectively).

It has been reported that the ion mitigation has strong correlation with the J-V hysteresis[4,5]. As shown in Supplementary Fig.12, forward and reverse scans were plotted at fresh and activated status, respectively.

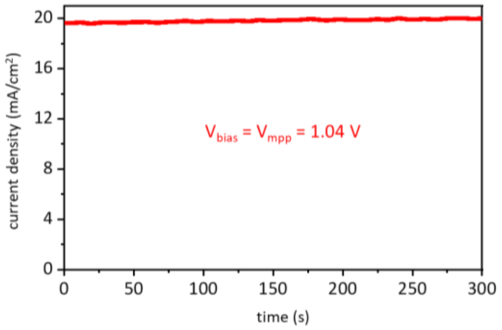

Supplementary Fig.13: current under bias versus time plot of the tT-phase solar cell

The photocurrent is tracked under the voltage at maximum power point (1.04 V) for 300 seconds under one-sun illumination, it is found that the photocurrent is slightly increased during the measurement.

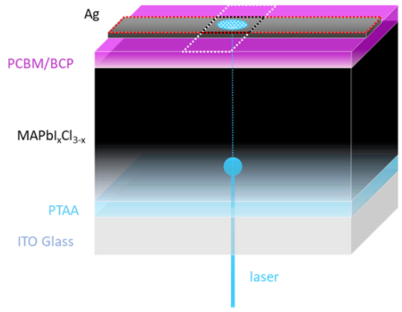

Supplementary Fig.14: schematic layout of sample showing the position of PL imaging, where white, red, black dotted lines mark area of transparent front contact, metallic back contact, and active area, respectively.

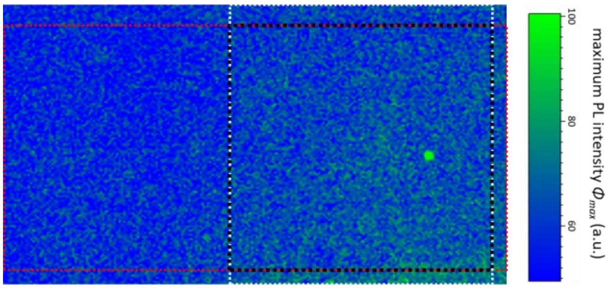

Supplementary Fig.15: PL imaging of T-phase $MAPbI_xCl_{3-x}$ solar cell after activation.

The PL imaging of T-phase $MAPbI_xCl_{3-x}$ solar cells after electric activation, the measurement method is the same as that for the tT-phase, the left and right parts are active area and dead area, respectively. The observation indicates a negligible difference between active and dead areas after activation. These results match with the J-V measurement, as we had observed no enhancement on photovoltaic performance after activation from T-phase solar cell. To be very specific there is no impact on the carrier life time and non-radiative recombination of the T-phase device with activation.

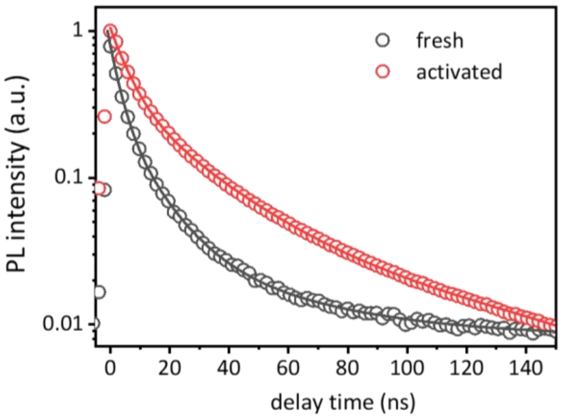

Supplementary Fig.16: transient PL of tT-phase solar cell before and after the activation, scatter dots and solid lines present measured data and fitted data, respectively.

Transient PL (tr-PL) measurement of tT-phase solar cells. The lifetimes increase for longer times and saturate for long times in the tens nanosecond range for both cases. The measured data is fitted using the following equation:

$$I_{(t)} = \sum_i A_i e^{-\frac{t}{\tau_i}}, (i \; = \; 1, 2, 3)$$

where A_i is the amplitude of each component, and τ_i is the corresponding lifetime. As summarized in Supplementary Table3, the tr-PL shows multiple-decay components, including a fast initial drop of intensity (within tens of ns) followed by a slow (hundreds of ns) relaxation process, carrier lifetime of the two major components is distinctly different (a few ns vs tens of ns). The multi-decay process implies a heterogeneous distribution of carriers with various lifetime values, they can be considered as two subpopulations of carriers with different recombination rates. The percentage of the long (short) lifetime carriers increased (decreased) after activation, indicating the recombination route is slightly varied after the activation.

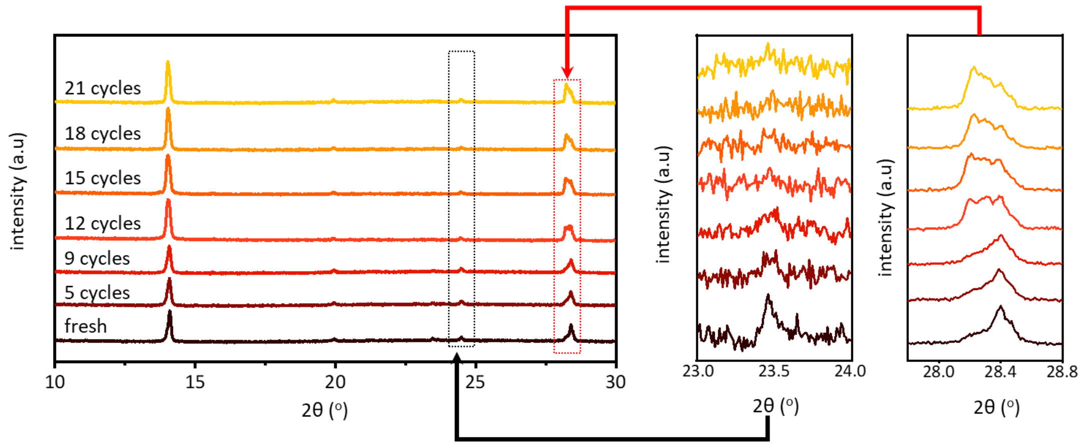

Supplementary Fig.17: XRD spectrum of tT-phase solar cells that have been activated for various cycles.

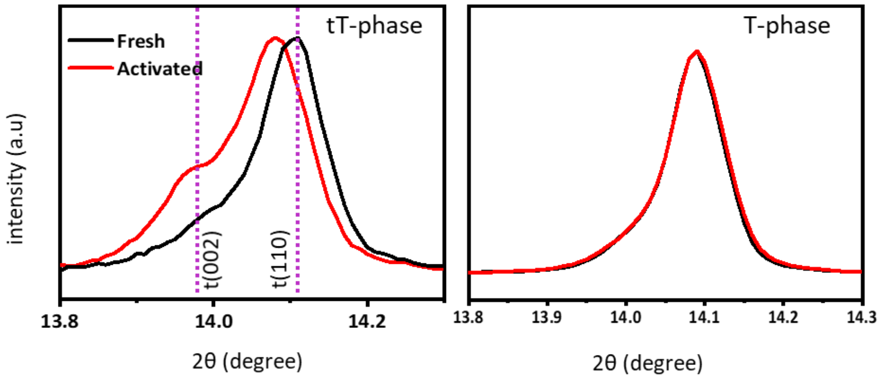

Supplementary Fig.18: XRD pattern of (a) tT-phase and (b) T-phase solar cells before and after electric activation.

The correlation between the crystallographic variation and photovoltaic performance was developed to understand the electric activation effect. As shown in Supplementary Fig.18, the XRD peak of tT-phase device shifted from 2θ=14.11° to 2θ=14.07°, along with the variation in the intensity of the t(002) peak. In contrast, no difference was observed in the peak position and intensity of the T-phase device. The results represent that upon activation, lattice parameters of the tT-phase expanded, and t(002) domains switched in a particular direction.

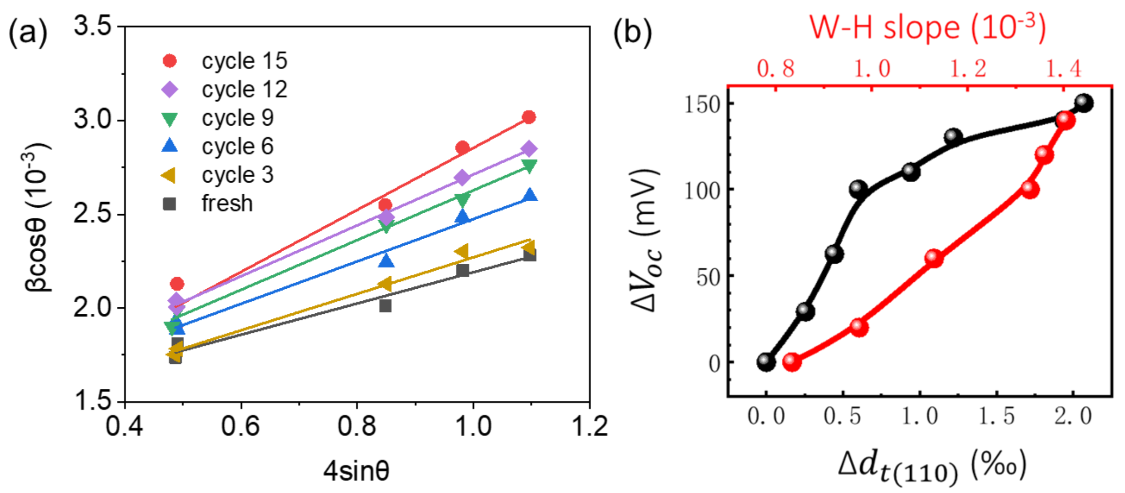

Supplementary Fig.19: (a). W-H slope along with the activation cycles. (b) V_{oc} enhancement versus two-types of crystallographic deformations along with the activation: interspacing and microstrain.

$$\Delta V_{oc} = V_{oc} - V_{oc,fresh} \text{ and } \Delta d_{t(110)} = (d_{t(110),activated} - d_{t(110),fresh}) \, / \, d_{t(110),fresh}$$

It is found that the V_{oc} is enhanced along with the elevated interspacing, the 2‰ increment of d corresponds to an V_{oc} enhancement of 150 mV.

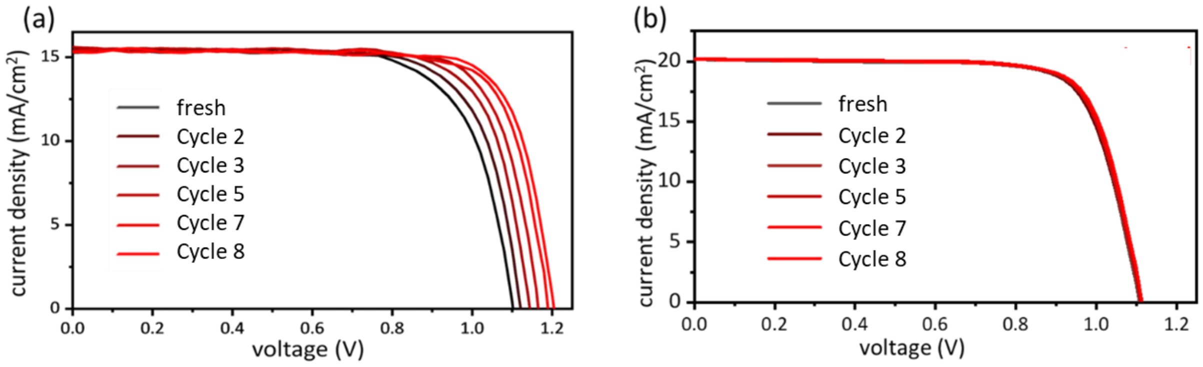

Supplementary Fig.20: current density versus voltage curves of tT-phase devices activated by (a) Electric field only method and (b) Illumination only method

It is found that the electric field can activate the solar cell without the illumination (Supplementary Fig.20a). On the contrary, no sign of V_{oc} or PCE increment can be observed only by illumination (Supplementary Fig.20b). The observation indicates that, even under illumination, if the device is short circuited and hence no electric field is applied , the device cannot be activated. The observation revealed that the electric field applied on $MAPbI_xCl_{3-x}$ is the trigger of activation effect, instead of the illumination.

Supplementary Table1: Photovoltaic performances of 0.04 cm^2 devices before and after the activation

device	fresh				activated			
	V_{oc} (V)	J_{sc} (mA/cm^2)	*FF* (%)	*PCE* (%)	V_{oc} (V)	J_{sc} (mA/cm^2)	*FF* (%)	*PCE* (%)
#1	1.11	19.93	78.77	17.43	1.24	20.15	81.32	20.25
#2	1.11	20.41	78.36	17.81	1.24	20.29	81.41	20.57
#3	1.12	20.30	77.60	17.59	1.24	19.34	80.89	19.44
#4	1.12	20.23	78.00	17.60	1.25	19.13	80.13	19.09
#5	1.10	19.41	73.57	15.63	1.25	19.13	78.27	19.13
#6	1.10	18.71	77.57	16.00	1.21	18.77	79.40	18.77
#7	1.12	20.54	76.18	17.64	1.23	19.05	79.15	19.05
#8	1.10	20.55	75.76	17.13	1.24	20.27	79.01	20.27
#9	1.10	19.38	76.46	16.33	1.23	19.19	80.79	19.19
#10	1.19	21.11	80.36	20.19	1.21	19.77	80.23	19.77
#11	1.10	19.58	76.73	17.17	1.22	19.09	80.71	19.09
#12	1.10	19.44	76.61	16.95	1.22	19.06	81.66	19.06
#13	1.10	19.03	75.71	16.37	1.21	19.53	80.32	19.53

Supplementary Table2: Photovoltaic performances of 1.0 cm^2 devices before and after activation

device	fresh				activated			
	V_{oc} (V)	J_{sc} (mA/cm^2)	*FF* (%)	*PCE* (%)	V_{oc} (V)	J_{sc} (mA/cm^2)	*FF* (%)	*PCE* (%)
#1	1.10	16.71	73.20	13.53	1.19	16.58	76.29	15.02
#2	1.10	17.71	73.22	14.26	1.21	17.33	80.52	16.96
#3	1.11	17.42	73.18	14.15	1.22	17.04	79.00	16.52
#4	1.11	16.35	75.16	13.67	1.21	16.10	78.72	15.43

Supplementary Table3: Fitting result of transient PL measurement

sample	A_1(%)	τ_1 (ns)	A_2(%)	τ_2 (ns)	A_3(%)	τ_3 (ns)	$\tau_{average}$ (ns)
fresh	66.18	3.26	29.79	11.01	4.02	40.09	7.05
activated	63.58	5.68	31.24	24.05	5.2	287.96	26.05

Supplementary Table4: Photovoltaic and crystallographic parameters of tT-phase devices along with the activated

activation (cycles)	V_{oc} (V)	*PCE* (%)	$A_{t(002)}$ / $A_{t(110)}$	$d_{t(110)}$ (Å)	$d_{t(002)}$ (Å)
fresh	1.14	15.48	0.29	6.278	6.302
5	1.18	15.92	0.65	6.280	6.309
9	1.22	16.86	0.76	6.282	6.318
12	1.24	17.03	0.89	6.286	6.322
15	1.26	18.40	0.93	6.291	6.328

Supplementary References

1 Rothmann, M. U., Li, W., Zhu, Y., Bach, U., Spiccia, L., Etheridge, J. & Cheng, Y.-B. Direct observation of intrinsic twin domains in tetragonal CH3NH3PbI3. *Nature Communications* **8**, 14547, doi:10.1038/ncomms14547 (2017).

2 Kennard, R. M., Dahlman, C. J., DeCrescent, R. A., Schuller, J. A., Mukherjee, K., Seshadri, R. & Chabinyc, M. L. Ferroelastic Hysteresis in Thin Films of Methylammonium Lead Iodide. *Chemistry of Materials* **33**, 298-309, doi:10.1021/acs.chemmater.0c03776 (2021).

3 Medjahed, A. A., Dally, P., Zhou, T., Lemaitre, N., Djurado, D., Reiss, P. & Pouget, S. Unraveling the Formation Mechanism and Ferroelastic Behavior of MAPbI3 Perovskite Thin Films Prepared in the Presence of Chloride. *Chemistry of Materials* **32**, 3346-3357, doi:10.1021/acs.chemmater.9b04239 (2020).

4 Zhang, T., Chen, H., Bai, Y., Xiao, S., Zhu, L., Hu, C., Xue, Q. & Yang, S. Understanding the relationship between ion migration and the anomalous hysteresis in high-efficiency perovskite solar cells: A fresh perspective from halide substitution. *Nano Energy* **26**, 620-630 (2016).

5 Huang, J.-Y., Chang, E.-W. & Wu, Y.-R. in *Proceedings of Asia-Pacific International Conference on Perovskite, Organic Photovoltaics and Optoelectronics (IPEROP20).*
