# Supplementary material for: Suppressing non-radiative recombination in metal halide perovskite solar cells by synergistic effect of ferroelasticity
Source: Nat Commun. 2023 Jan 17;14:256. doi: 10.1038/s41467-023-35837-1 (PMC9845300; doi:10.1038/s41467-023-35837-1)
Supplement: Supplementary file 2 — Solar Cells Reporting Summary [file 41467_2023_35837_MOESM2_ESM.pdf]

nature research

Corresponding author(s): Can Li

Solar Cells Reporting Summary

Nature Research wishes to improve the reproducibility of the work that we publish. This form is intended for publication with all accepted papers reporting the characterization of photovoltaic devices and provides structure for consistency and transparency in reporting. Some list items might not apply to an individual manuscript, but all fields must be completed for clarity.

For further information on Nature Research policies, including our data availability policy, see Authors & Referees.

Experimental design

Please check: are the following details reported in the manuscript?

1. Dimensions

Item	Yes	No	Details
Area of the tested solar cells	[x]	[ ]	See "Device performance measurement" sub-section, page16 of manuscript.
Method used to determine the device area	[x]	[ ]	See "Device performance measurement" sub-section, page16 of manuscript.

2. Current-voltage characterization

Item	Yes	No	Details
Current density-voltage (J-V) plots in both forward and backward direction	[x]	[ ]	See Supplementary Fig.10 and Supplementary Fig.12 in supporting information.
Voltage scan conditions<br>*For instance: scan direction, speed, dwell times*	[x]	[ ]	See "Device performance measurement" sub-section, page16 of manuscript.
Test environment<br>*For instance: characterization temperature, in air or in glove box*	[x]	[ ]	See "Device performance measurement" sub-section, page16 of manuscript.
Protocol for preconditioning of the device before its characterization	[x]	[ ]	See "Electric activation process" sub-section, page16 of manuscript.
Stability of the J-V characteristic<br>*Verified with time evolution of the maximum power point or with the photocurrent at maximum power point; see ref. 7 for details.*	[x]	[ ]	See Supplementary Fig.13 in supporting information.

3. Hysteresis or any other unusual behaviour

Item	Yes	No	Details
Description of the unusual behaviour observed during the characterization	[x]	[ ]	1. The PCE of the tT-phase device is found increased under electric activation.<br>2. The increase shows no drop or degradation after the long-term monitor.
Related experimental data	[x]	[ ]	Figure 2a, 2b, 2c, 3a, 3f in the manuscript; Supplementary Fig.10, 11, 12, 20 and Table 1,2,4 in the supporting information.

4. Efficiency

Item	Yes	No	Details
External quantum efficiency (EQE) or incident photons to current efficiency (IPCE)	[x]	[ ]	See Supplementary Fig.9 in supporting information.
A comparison between the integrated response under the standard reference spectrum and the response measure under the simulator	[ ]	[x]	Not relevant to the core of this study, that is the Voc and FF increase under the activation effect.
For tandem solar cells, the bias illumination and bias voltage used for each subcell	[ ]	[x]	No tandem solar cells involved in this study.

5. Calibration

Item	Yes	No	Details
Light source and reference cell or sensor used for the characterization	[x]	[ ]	See "Device performance measurement" sub-section, page16 of manuscript
Confirmation that the reference cell was calibrated and certified	[x]	[ ]	See "Device performance measurement" sub-section, page16 of manuscript

November 2017

Calculation of spectral mismatch between the reference cell and the devices under test	☐ Yes ☒ No	calculation is not reported but we reported all needed information including EQE and the calibration of solar simulator ("Device performance measurement" sub-section, page16 of supporting information).

6. Mask/aperture

Size of the mask/aperture used during testing	☐ Yes ☒ No	no mask used during the measurement.
Variation of the measured short-circuit current density with the mask/aperture area	☐ Yes ☒ No	no mask used during the measurement.

7. Performance certification

Identity of the independent certification laboratory that confirmed the photovoltaic performance	☐ Yes ☒ No	not relevant to the core of this study.
A copy of any certificate(s)<br>*Provide in Supplementary Information*	☐ Yes ☒ No	not relevant to the core of this study.

8. Statistics

Number of solar cells tested	☒ Yes ☐ No	caption of Figure 2b, 23 devices.
Statistical analysis of the device performance	☒ Yes ☐ No	results of statistic analysis is presented in Figure 2b.

9. Long-term stability analysis

Type of analysis, bias conditions and environmental conditions<br>*For instance: illumination type, temperature, atmosphere humidity, encapsulation method, preconditioning temperature*	☒ Yes ☐ No	results of long-term stability analysis is presented in Figure 2c.
