# Supplementary figures and images for: Suppressing non-radiative recombination in metal halide perovskite solar cells by synergistic effect of ferroelasticity
Source: Nat Commun. 2023 Jan 17;14:256. doi: 10.1038/s41467-023-35837-1 (PMC9845300; doi:10.1038/s41467-023-35837-1)

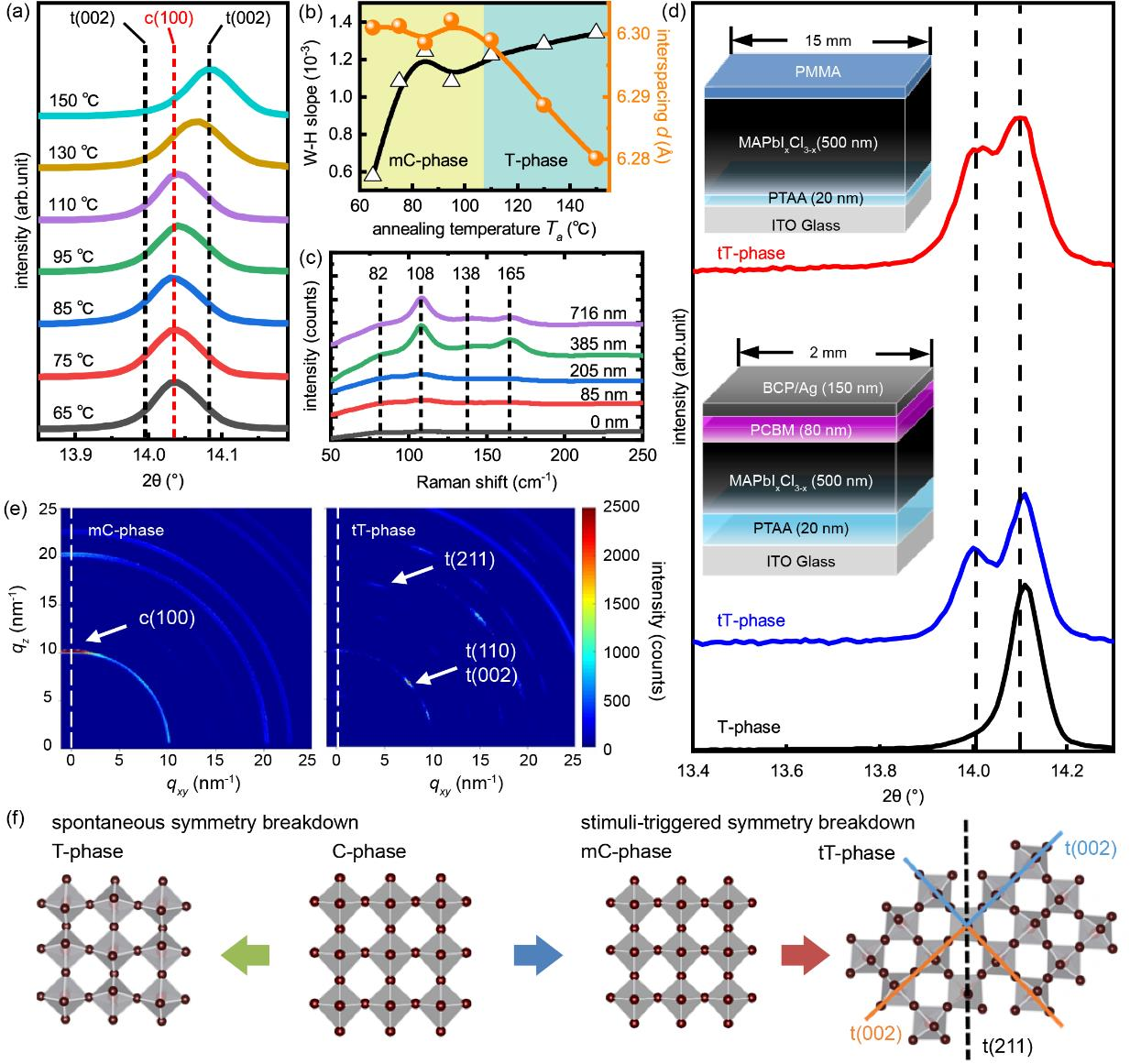

Supplement: Supplementary file 3 — Source Data [file 41467_2023_35837_MOESM3_ESM.zip › All figures/Figure-01/1.tif]

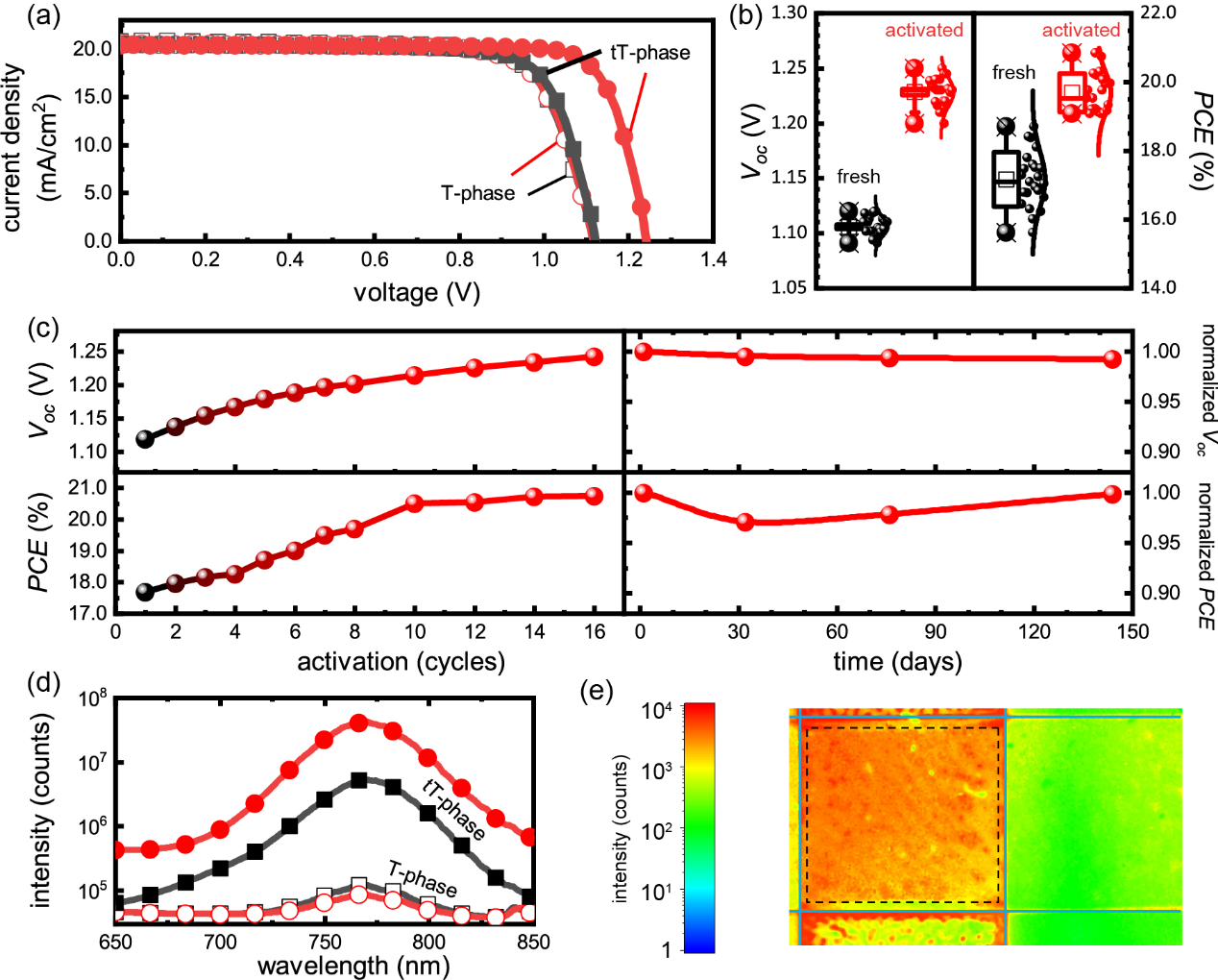

Supplement: Supplementary file 3 — Source Data [file 41467_2023_35837_MOESM3_ESM.zip › All figures/Figure-02/Fig-02.tif]

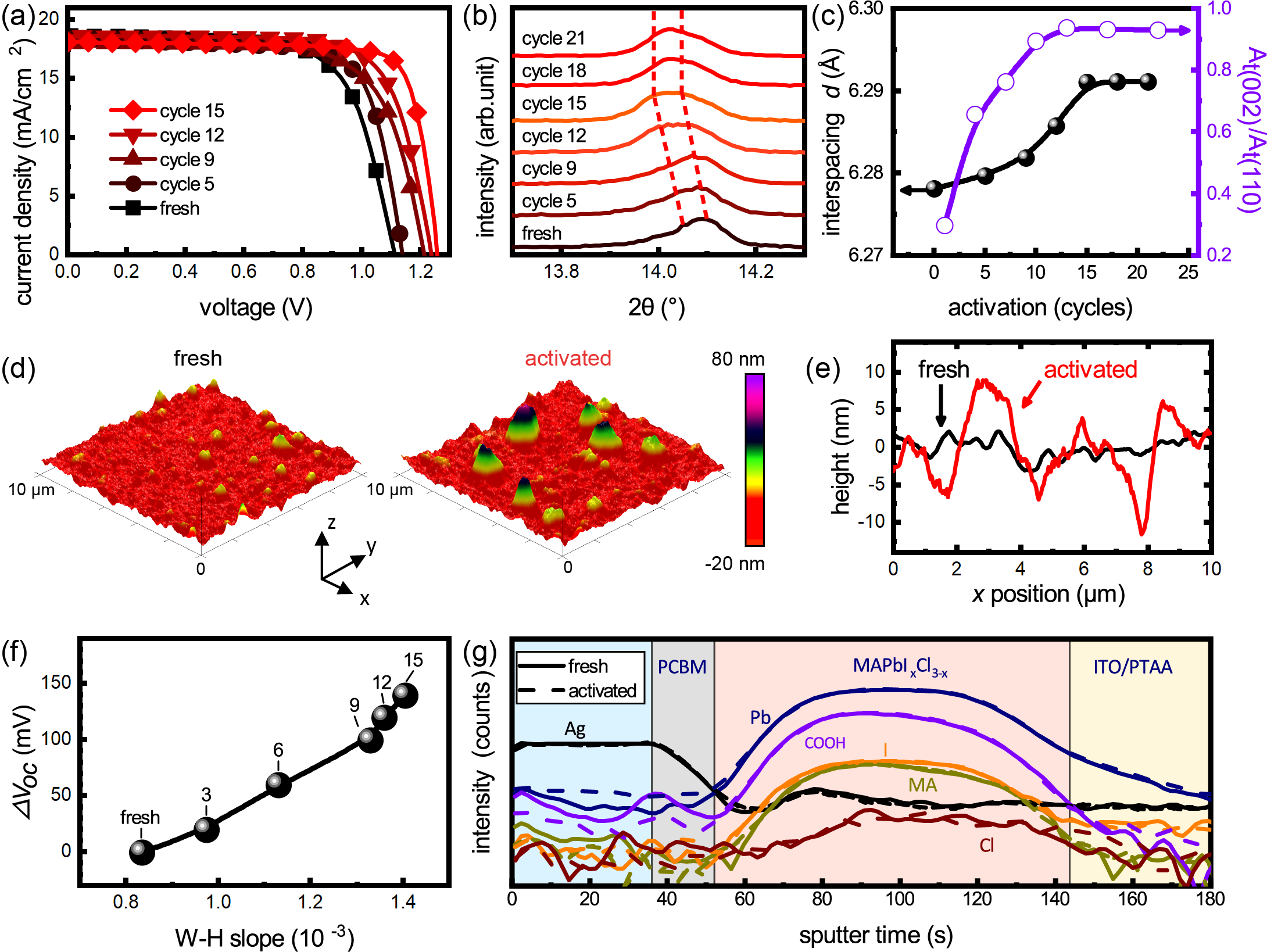

Supplement: Supplementary file 3 — Source Data [file 41467_2023_35837_MOESM3_ESM.zip › All figures/Figure-03/03.tif]

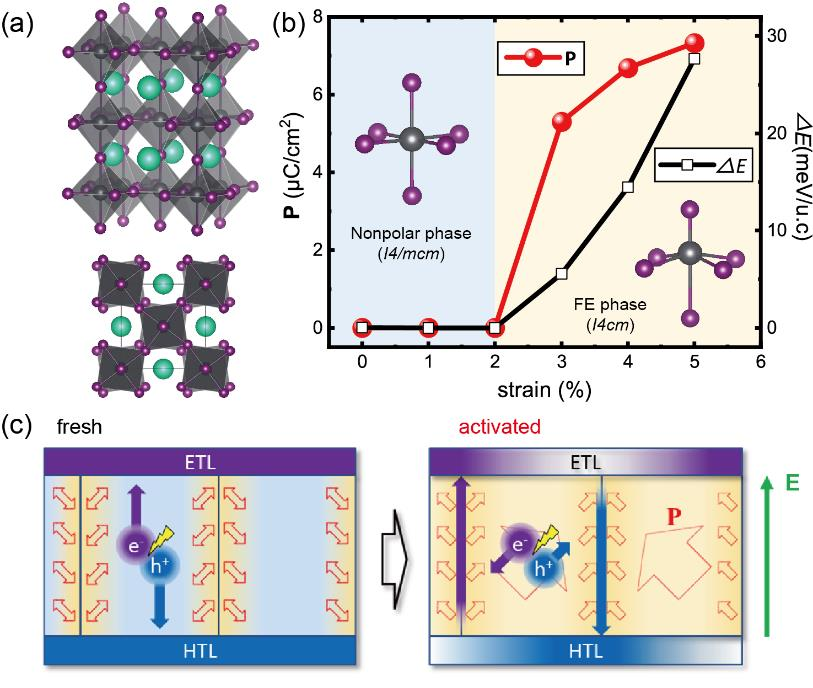

Supplement: Supplementary file 3 — Source Data [file 41467_2023_35837_MOESM3_ESM.zip › All figures/Figure-04/Fig-04.tif]

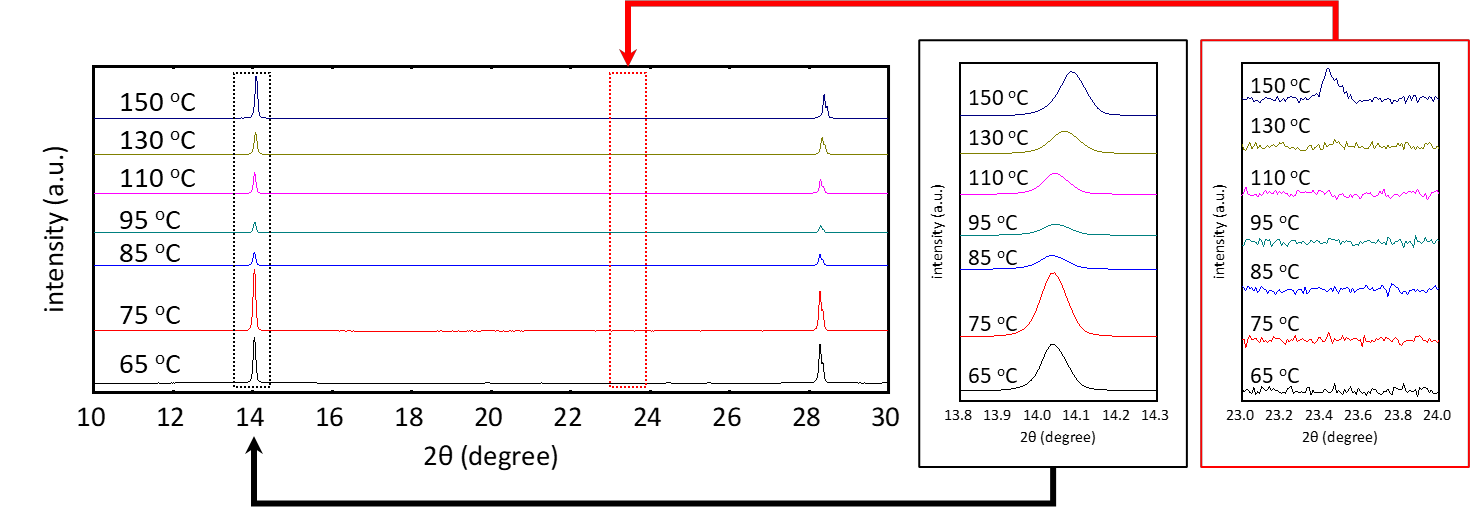

Supplement: Supplementary file 3 — Source Data [file 41467_2023_35837_MOESM3_ESM.zip › All figures/Figure-S1/S1.tif]

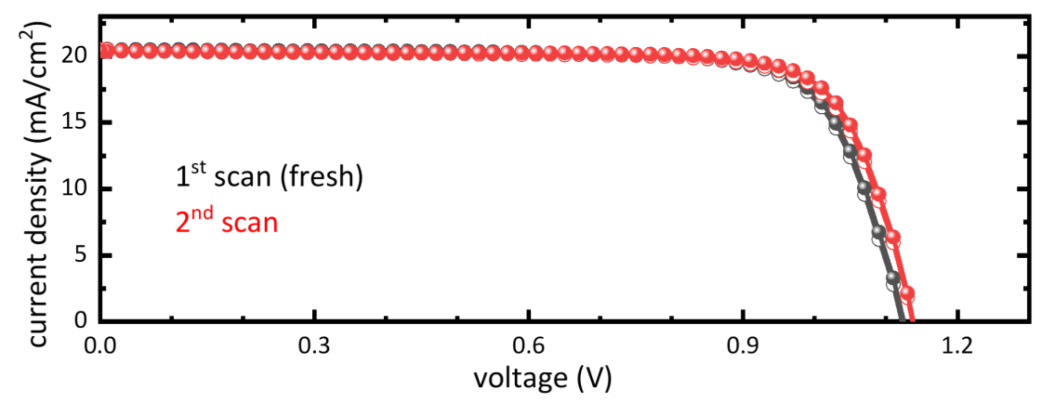

Supplement: Supplementary file 3 — Source Data [file 41467_2023_35837_MOESM3_ESM.zip › All figures/Figure-S10/10.tif]

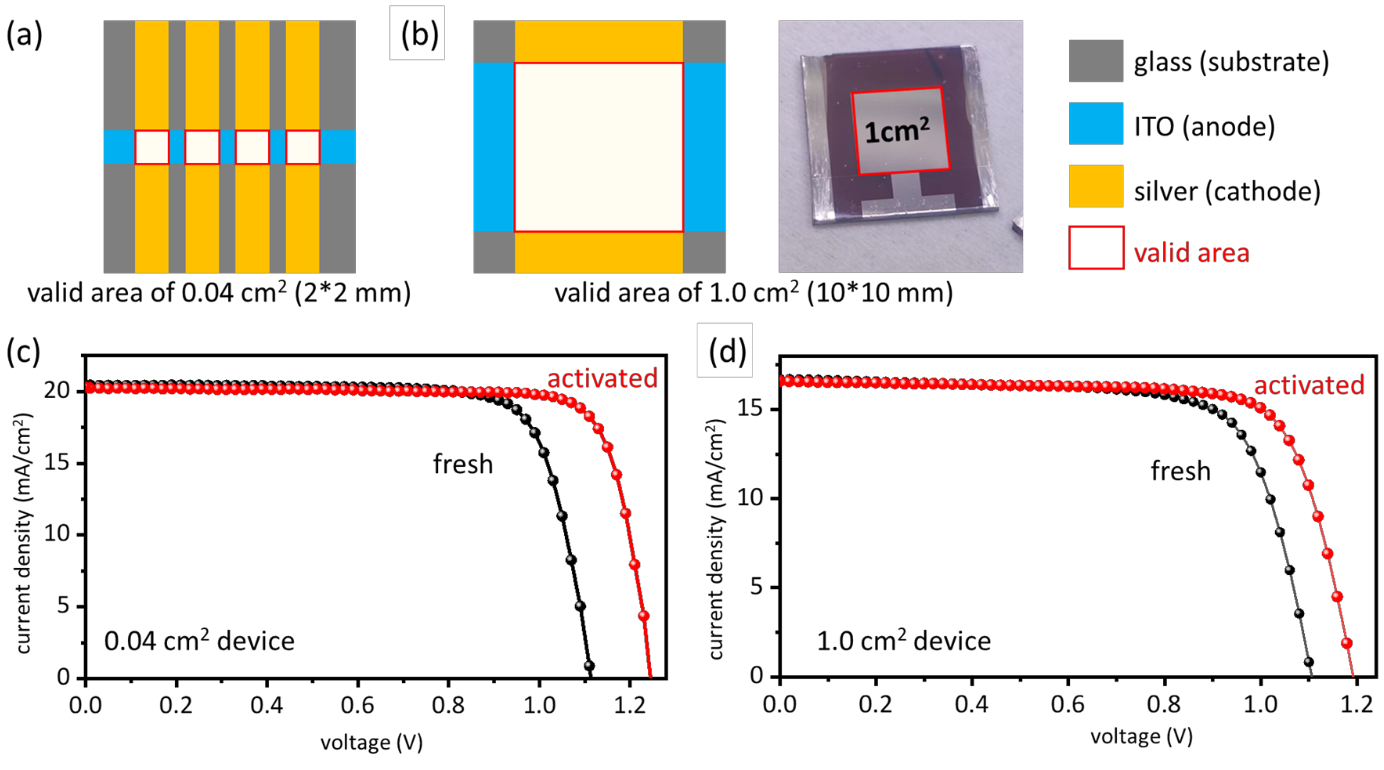

Supplement: Supplementary file 3 — Source Data [file 41467_2023_35837_MOESM3_ESM.zip › All figures/Figure-S11/11.tif]

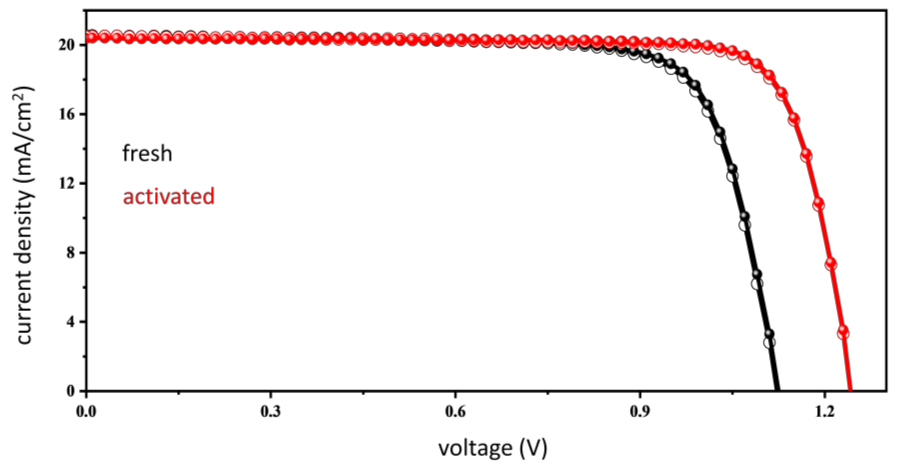

Supplement: Supplementary file 3 — Source Data [file 41467_2023_35837_MOESM3_ESM.zip › All figures/Figure-S12/12.tif]

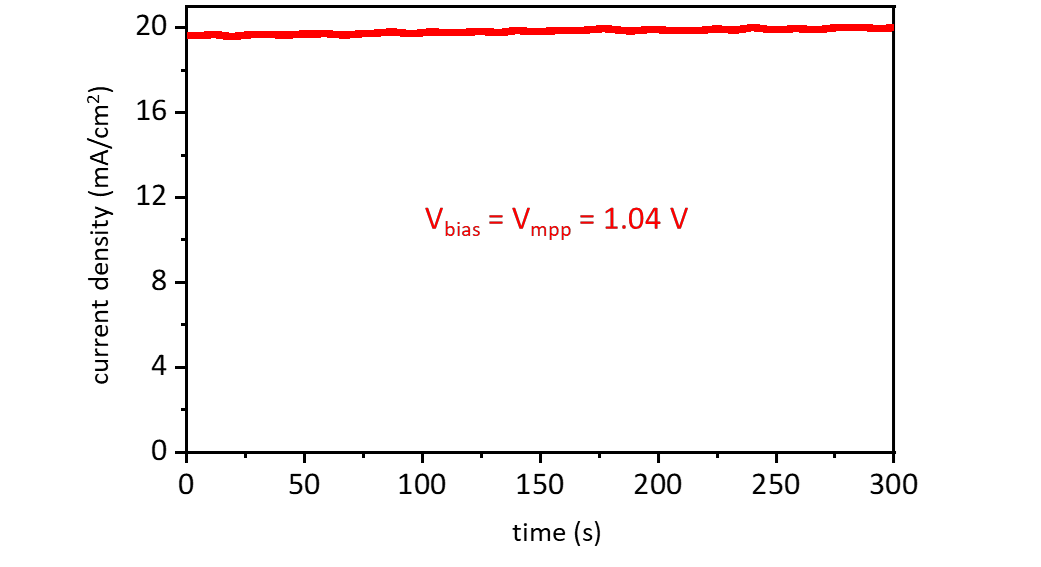

Supplement: Supplementary file 3 — Source Data [file 41467_2023_35837_MOESM3_ESM.zip › All figures/Figure-S13/13.tif]

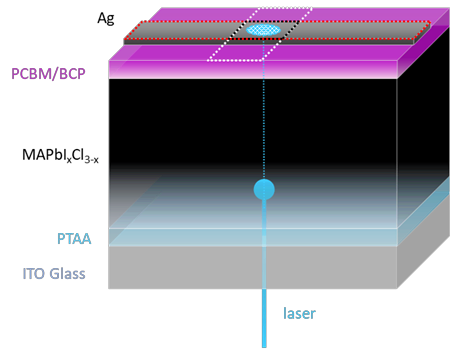

Supplement: Supplementary file 3 — Source Data [file 41467_2023_35837_MOESM3_ESM.zip › All figures/Figure-S14/14.tif]

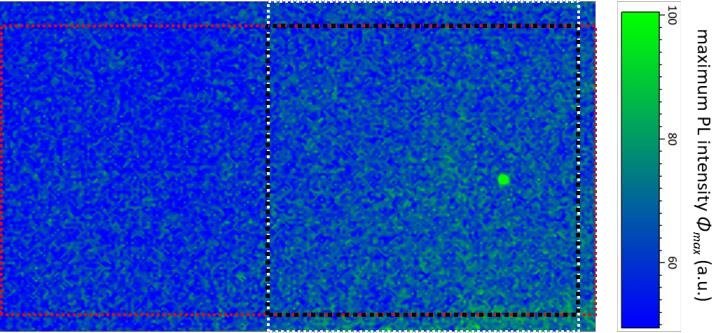

Supplement: Supplementary file 3 — Source Data [file 41467_2023_35837_MOESM3_ESM.zip › All figures/Figure-S15/15.tif]

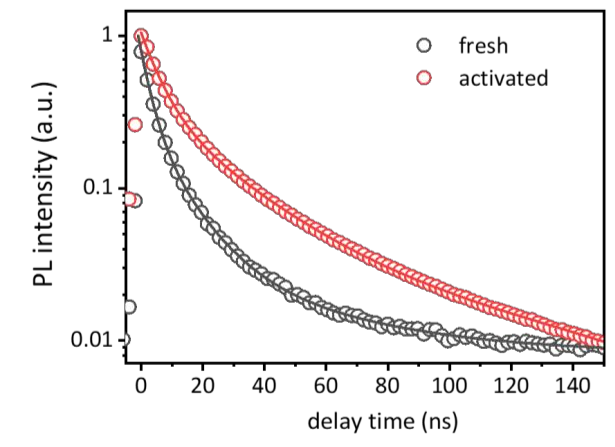

Supplement: Supplementary file 3 — Source Data [file 41467_2023_35837_MOESM3_ESM.zip › All figures/Figure-S16/16.tif]

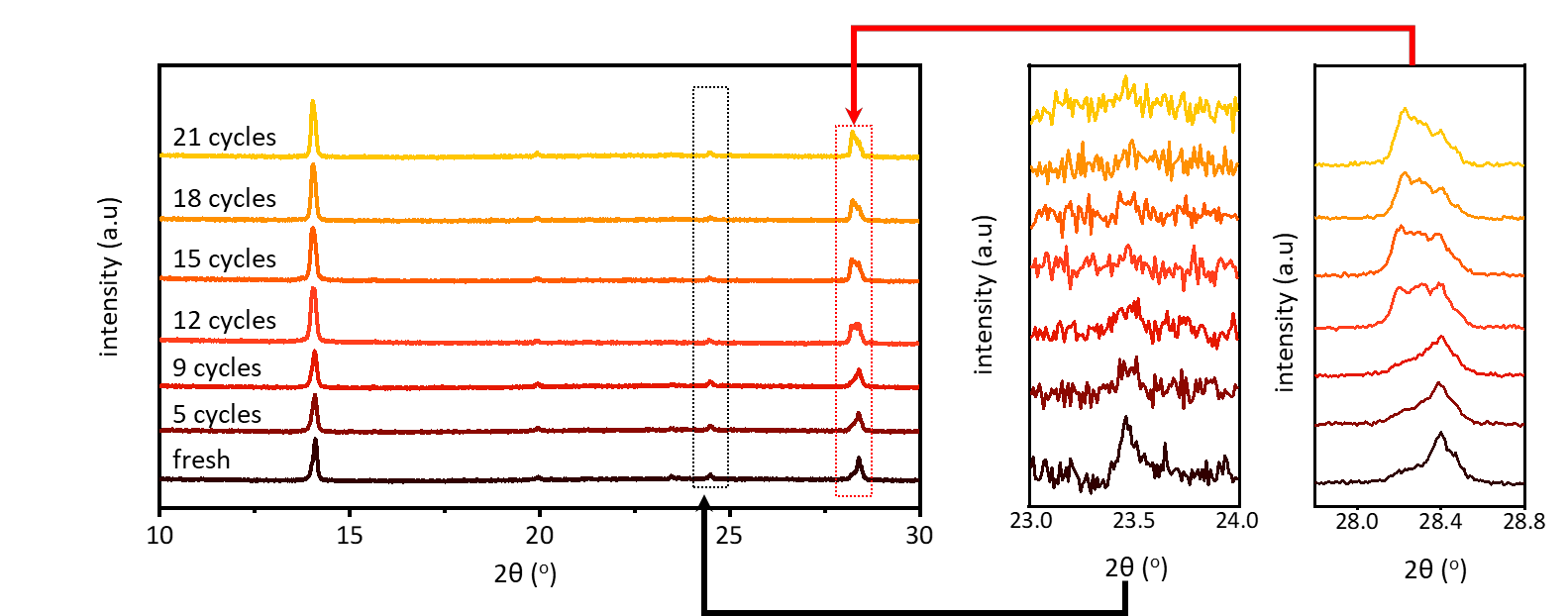

Supplement: Supplementary file 3 — Source Data [file 41467_2023_35837_MOESM3_ESM.zip › All figures/Figure-S17/17.tif]

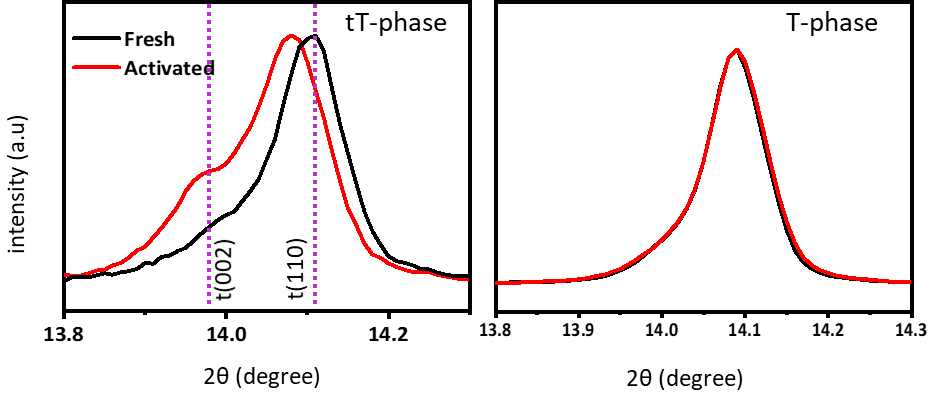

Supplement: Supplementary file 3 — Source Data [file 41467_2023_35837_MOESM3_ESM.zip › All figures/Figure-S18/18.tif]

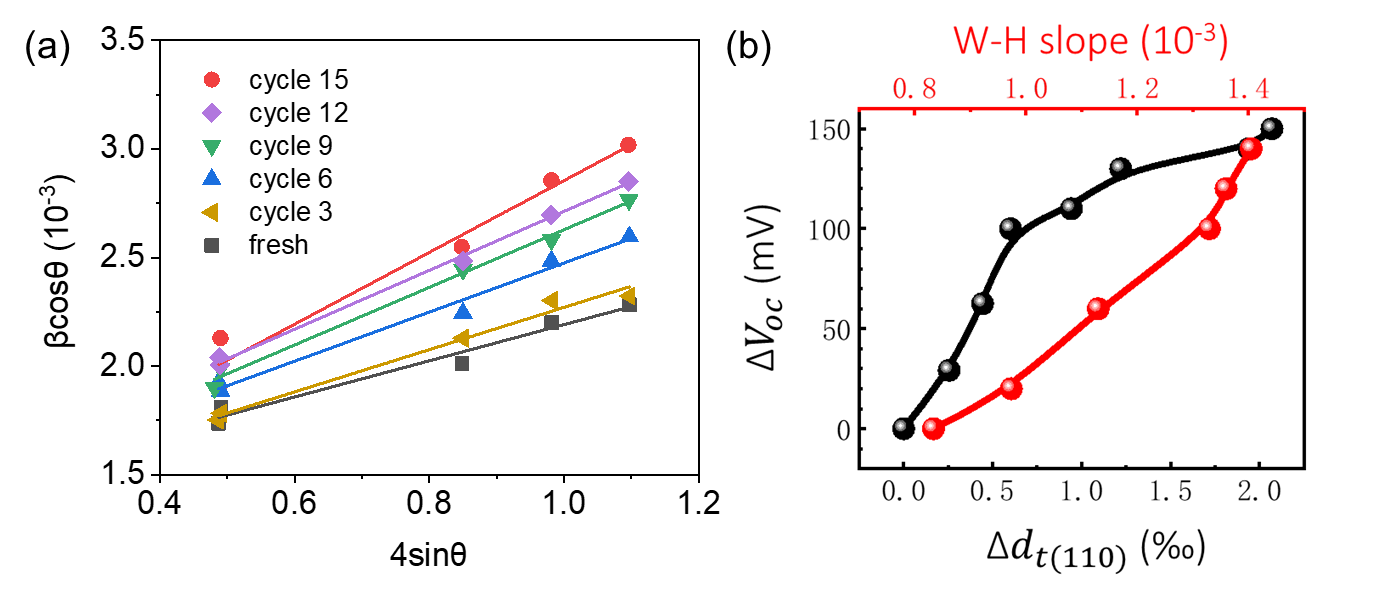

Supplement: Supplementary file 3 — Source Data [file 41467_2023_35837_MOESM3_ESM.zip › All figures/Figure-S19/19.tif]

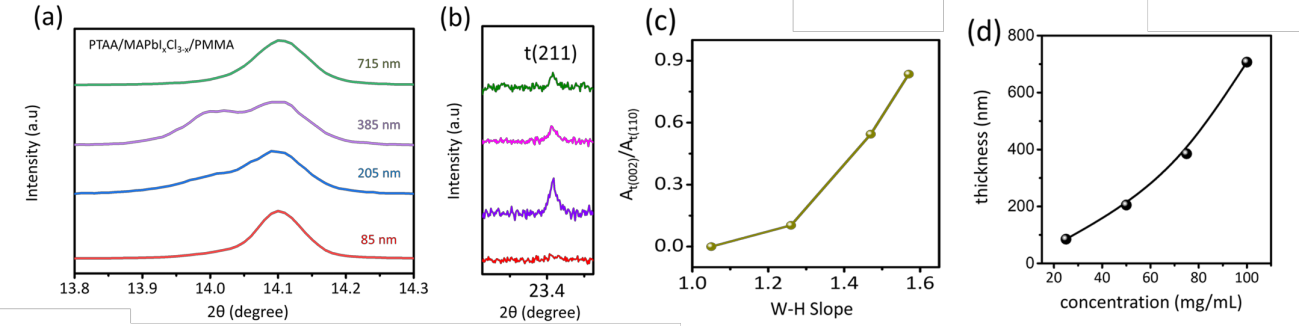

Supplement: Supplementary file 3 — Source Data [file 41467_2023_35837_MOESM3_ESM.zip › All figures/Figure-S2/S2.tif]

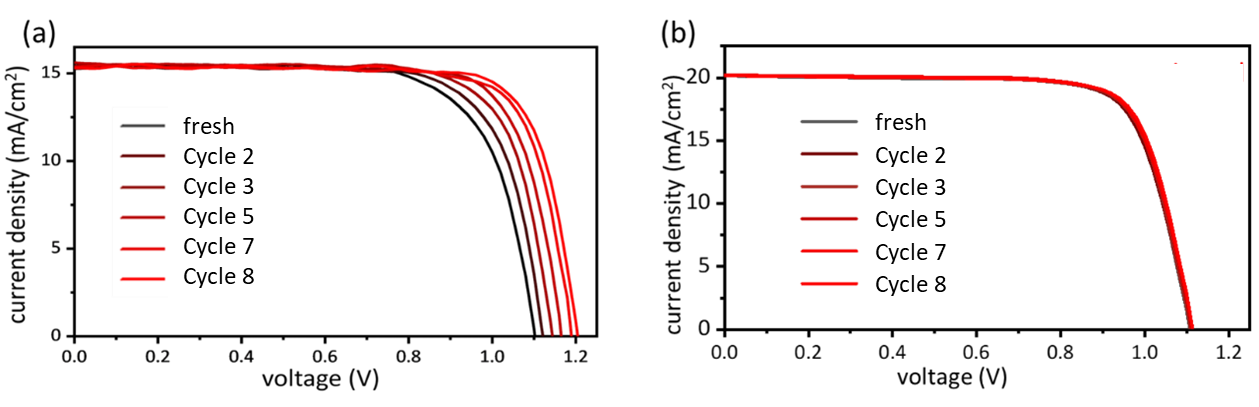

Supplement: Supplementary file 3 — Source Data [file 41467_2023_35837_MOESM3_ESM.zip › All figures/Figure-S20/20.tif]

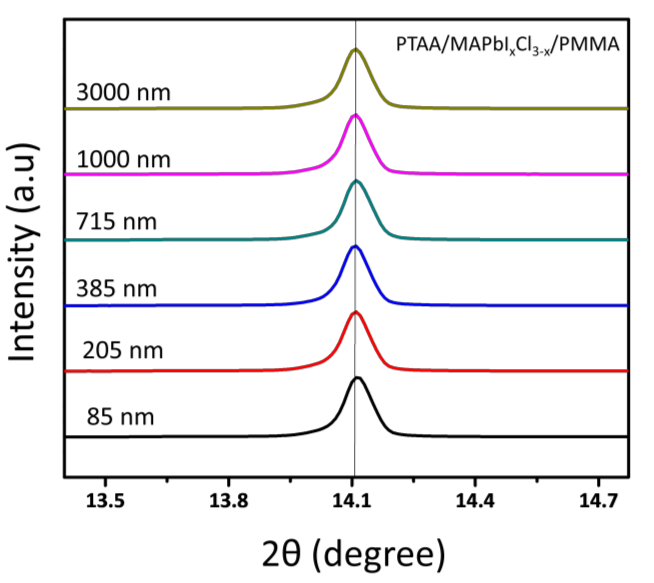

Supplement: Supplementary file 3 — Source Data [file 41467_2023_35837_MOESM3_ESM.zip › All figures/Figure-S3/3.tif]

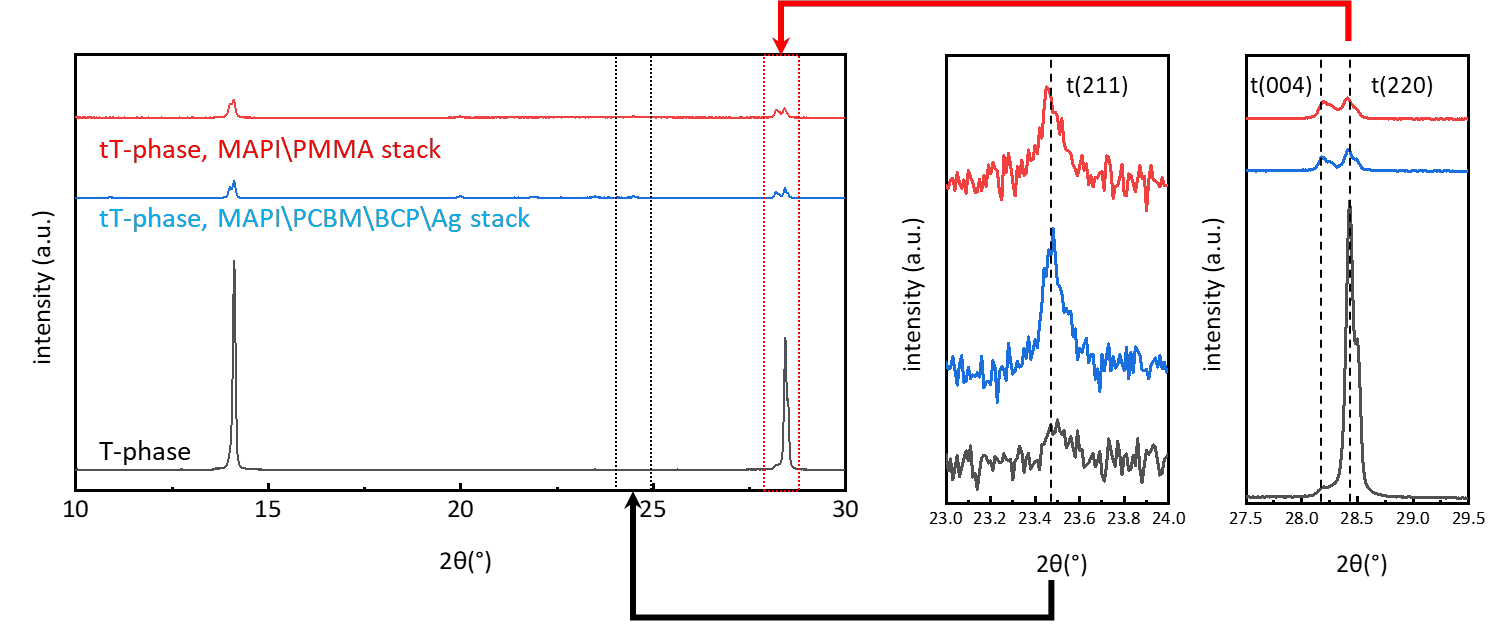

Supplement: Supplementary file 3 — Source Data [file 41467_2023_35837_MOESM3_ESM.zip › All figures/Figure-S4/4.tif]

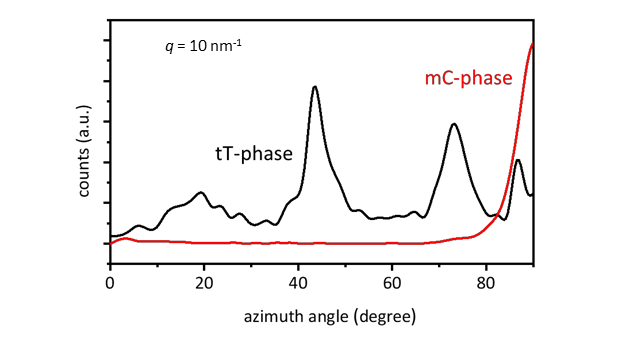

Supplement: Supplementary file 3 — Source Data [file 41467_2023_35837_MOESM3_ESM.zip › All figures/Figure-S5/5.tif]

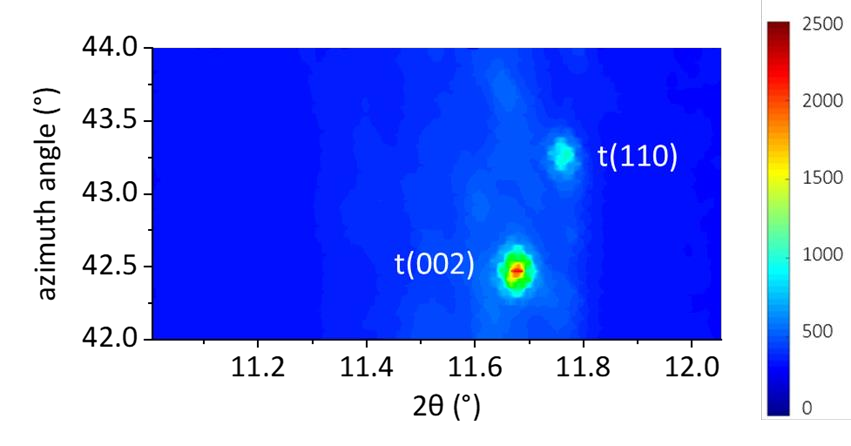

Supplement: Supplementary file 3 — Source Data [file 41467_2023_35837_MOESM3_ESM.zip › All figures/Figure-S6/6.tif]

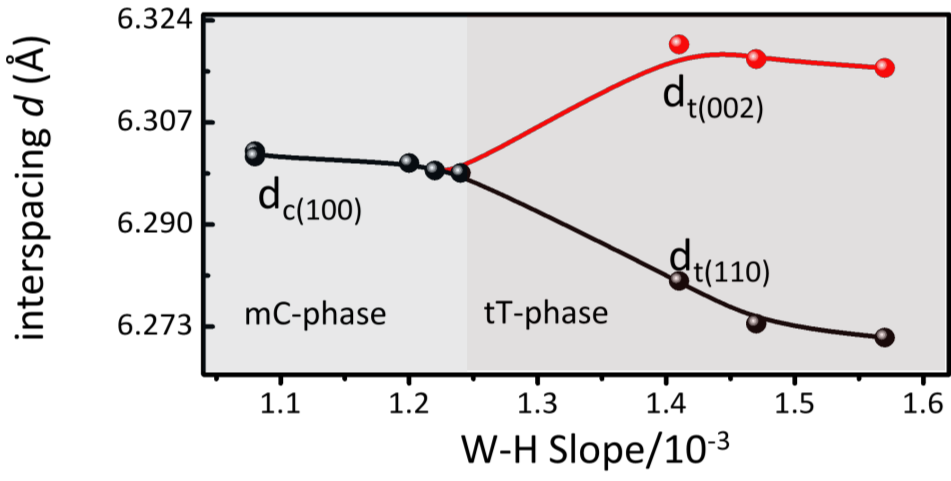

Supplement: Supplementary file 3 — Source Data [file 41467_2023_35837_MOESM3_ESM.zip › All figures/Figure-S7/7.tif]

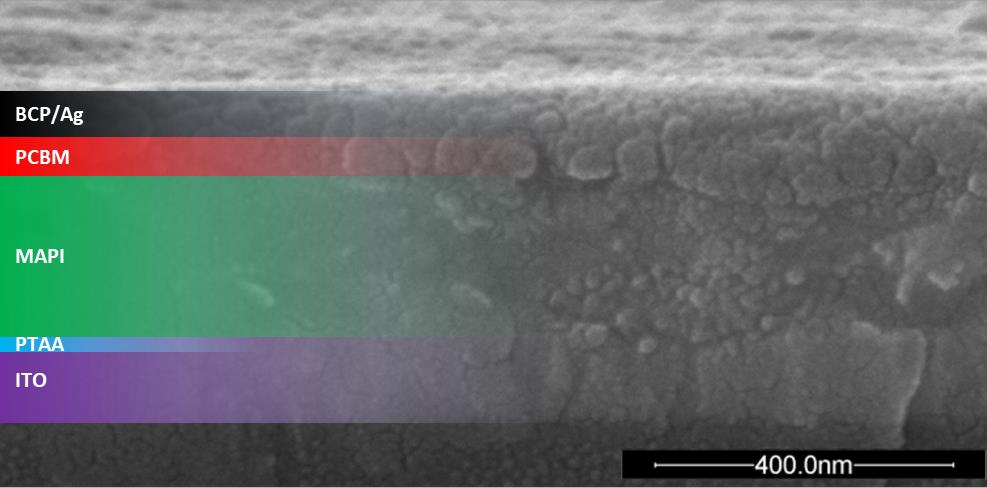

Supplement: Supplementary file 3 — Source Data [file 41467_2023_35837_MOESM3_ESM.zip › All figures/Figure-S8/8.tif]

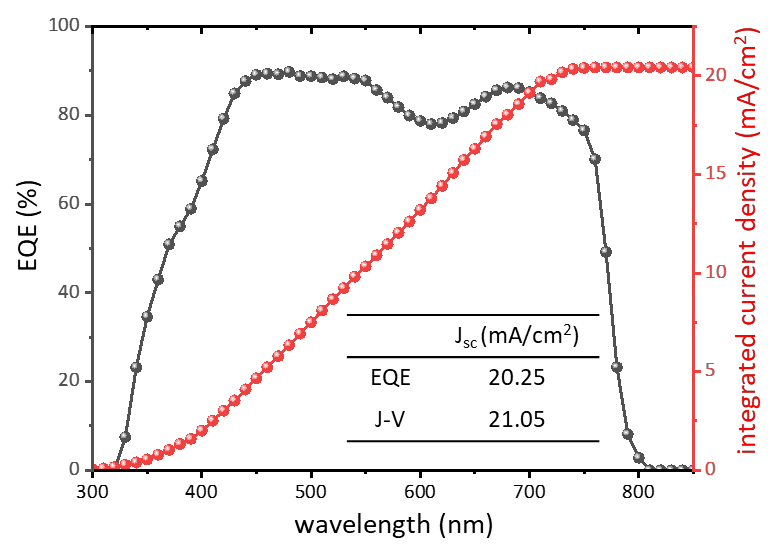

Supplement: Supplementary file 3 — Source Data [file 41467_2023_35837_MOESM3_ESM.zip › All figures/Figure-S9/9.tif]
